# Host Protein-Degrading Cathepsin L-like Proteases of *Ostertagia ostertagi*

**DOI:** 10.3390/ijms27209219

**Published:** 2026-10-17

**Authors:** Athira C. Karunakaran, Yaileen Aponte, Arunraj M. Rajendrakumar, Yuhui Liu, Clarissa Boschiero, Eliseo Miramontes, Ethiopia Beshah, Jonathan Shao, Bryan Vinyard, George E. Liu, Xiaoping Zhu, Wenbin Tuo

**Affiliations:** 1 Animal Parasitic Diseases Laboratory, Agricultural Research Service, U.S. Department of Agriculture, Beltsville, MD 20705, USA; athira.karunakaran@usda.gov (A.C.K.); yaponteb@purdue.edu (Y.A.); arunraj@umd.edu (A.M.R.); lucasliu6859@gmail.com (Y.L.); eliseo.miramontes@usda.gov (E.M.); ethiopia.beshah@usda.gov (E.B.); jonathan.shao@usda.gov (J.S.); 2 Oak Ridge Institute for Science and Education, Oak Ridge, TN 37830, USA; 3 Purdue University College of Veterinary Medicine, West Lafayette, IN 47907, USA; 4 Department of Veterinary Medicine, University of Maryland, College Park, MD 20740, USA; cboschi@umd.edu (C.B.); xzhu1@umd.edu (X.Z.); 5 Animal Genomics Improvement Laboratory, Agricultural Research Service, U.S. Department of Agriculture, Beltsville, MD 20705, USA; george.liu@usda.gov; 6 Statistics and Bioinformatics Group, Agricultural Research Service, United States Department of Agriculture, Beltsville, MD 20705, USA; bryan.vinyard@usda.gov

**Keywords:** gastrointestinal nematode, *Ostertagia ostertagi*, cathepsin, cysteine proteases, mucin, cattle

## Abstract

*Ostertagia ostertagi* is the most pathogenic gastrointestinal nematode of cattle and causes major productivity losses. With rising anthelmintic resistance and significant economic impact, alternative control measures, such as vaccination, are urgently needed. This study aimed to generate functionally active recombinant cysteine proteases from *O. ostertagi*, enzymes involved in nutrient acquisition and immune evasion, to evaluate their potential as vaccine candidates. Two phylogenetically predicted cathepsin B (catB)-like proteases, OoCBP1 and OoCBP2, were cloned into the pcDNA3.1+ vector and expressed in FreeStyle 293-F cells. Purified proteins were assessed using fluorogenic substrates and various host protein substrates, including fibrinogen, albumin, hemoglobin, IgG, mucin, and gelatin, at pH 5.0 and 7.4 with or without a reducing agent. OoCBP1 was detected in the parasite across the life stages, while OoCBP2 was only detectable in adult worms. OoCBP1 partially degraded host proteins at pH 5.0, with reduced activity at pH 7.4, whereas OoCBP2 efficiently degraded all tested proteins at both pH levels except IgG, which was only partially degraded. OoCBP1 showed catB/L activity only at acidic pH, while OoCBP2 was active at both pH conditions. Activities were inhibited by catB, catB/L, or other cysteine protease inhibitors. OoCBP1, but not OoCBP2, required low pH-mediated autoactivation for substrate degradation. OoCBP1 had no effect on coagulation under physiological conditions, while OoCBP2 accelerated clot formation with reduced clot density. With in vitro activation, both proteases delayed coagulation. These findings indicate that OoCBP1 and OoCBP2 may contribute to nutrient acquisition and immune evasion, supporting their potential as targets for vaccines or chemotherapeutics against ostertagiosis.

## 1. Introduction

*Ostertagia ostertagi*, commonly referred to as the brown stomach worm or medium stomach worm, is a highly pathogenic gastrointestinal (GI) nematode that causes clinical ostertagiosis, primarily affecting calves during their first grazing season and older cattle encountering their initial infection. Subclinical cases are mainly found in adults. Clinical signs include watery diarrhea, often accompanied by anorexia, dehydration, dull hair coats, and soiled hindquarters, with notable weight loss, as well as a reduction in milk yield, thus causing substantial economic losses to livestock industries [1,2,3,4].

*Ostertagia ostertagi* belongs to the phylum Nematoda, Clade V, order Strongylida, family Trichostrongylidae, and genus *Ostertagia* [5]. The life cycle of *O. ostertagi* comprises two stages: the free-living stage (pre-parasitic) occurring on pasture and the parasitic stage in the bovine abomasum, with a prepatent period of 3 weeks. Adult female worms release eggs that are expelled in the feces. These eggs undergo hatching on pasture into first-stage larvae (L1), which then molt into second-stage larvae (L2) and, ultimately, into infective third-stage larvae (L3), in ideal conditions, taking about 2 weeks. Cattle become infected by ingesting the L3 while grazing. Upon ingestion, the L3 shed their protective sheaths in the rumen and then move to the abomasum and enter the lumen of gastric glands, where they molt into fourth-stage larvae (L4). If development proceeds uninterrupted to the adult stages, the worms emerge from the gastric glands approximately three weeks after infection, causing a condition known as type I ostertagiosis. The L4 can enter a dormant state known as diapause or hypobiosis, characterized by a cessation of development that may last for months. The synchronized emergence of hypobiotic larvae from gastric glands weeks or months after infection causes type II ostertagiosis [6,7]. Both forms of ostertagiosis cause damage to the abomasal mucosa. These affected cells are eventually substituted with hyperplastic, undifferentiated cuboidal cells (discrete or confluent nodules) [8]. Consequently, in the event of heavy infections, the abomasal pH rises from 2.0 to greater than 6.0. From a clinical perspective, when the pH exceeds 4.5, digestion in the abomasum ceases, resulting in a protein-losing gastropathy marked by symptoms such as weight loss, reduced feed intake, impaired GI function, and altered protein, energy and mineral metabolism [1]. The immune response against *O. ostertagi* is weak and requires prolonged exposure before it becomes evident [9]. The control of *O. ostertagi* relies on anthelmintic treatments; however, there is a growing concern regarding the escalating resistance to macrocyclic lactones in the United States [10] across Europe, South America, New Zealand, and Oceania [11,12,13,14]. Considering the pathogenicity and significant economic ramifications compounded by increasing resistance to anthelmintic drugs associated with this parasite [15,16,17], there is a critical need to investigate alternative control strategies [18,19,20,21,22]. Although vaccination has yet to succeed, it remains a promising, eco-friendly option that targets both susceptible and resistant parasites. A better understanding of host–parasite interactions is essential for identifying new vaccine targets. One such promising avenue is the potential for vaccination targeting *Ostertagia* proteases present in excretory/secretory (ES) products [23]. Proteases are essential for the survival of these parasites, as they facilitate various functions such as immune evasion, nutrient acquisition, host penetration, and molting [19,24].

Indeed, vaccination against *Ostertagia* with antigens enriched in cysteine proteases induces a protective immune response with an 80% reduction in fecal egg counts [25]. Similarly, two cysteine proteases produced in the gut of adult schistosomes confer protection in a murine model [26,27,28]. Furthermore, vaccination with cathepsin L (catL) proteases in cattle reduces the viability of *Fasciola hepatica* eggs, preventing the transmission of liver fluke disease [29] and playing a role in the parasite’s immune evasion strategies [30,31]. In addition, cysteine proteases of *Haemonchus contortus*, such as catB-like proteins 1 and 2 (HcCBP1 and HcCBP2), modulate host immune responses, nutrition acquisition, and blood coagulation during infection [32,33].

In this study, we characterized two functionally active proteases, OoCBP1 and OoCBP2, orthologs of HcCBP1 and HcCBP2, which were demonstrated to be expressed in different developmental stages of *O. ostertagi*. We expressed these proteins in mammalian cells and tested their ability to degrade host proteins and synthetic substrates, as well as to regulate blood coagulation through fibrin polymerization and recalcification assays. The OoCBPs were shown to be catL-like proteases and degraded host protein substrates. The functions of these proteases may indicate their potential role in blood coagulation, nutrient acquisition and immune evasion, thereby contributing to the survivability of the parasite within the host.

## 2. Results

### 2.1. Sequence Analysis of Full-Length OoCBP1 and OoCBP2

**Figure 1:**
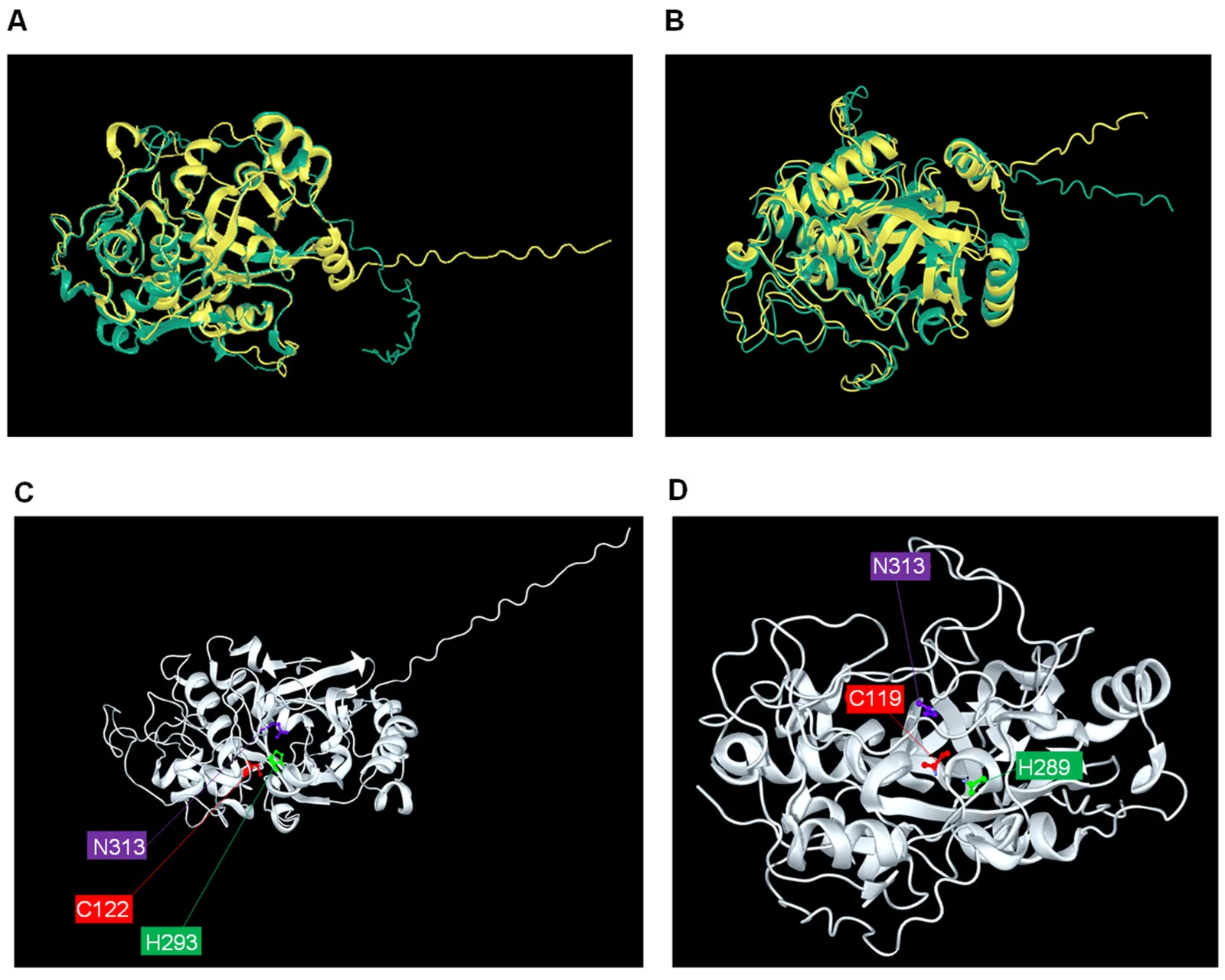
Structural alignments of *Haemonchus contortus* cysteine proteases (HcCBP1 and HcCBP2) with their *Ostertagia ostertagi* orthologues (OoCBP1 and OoCBP2) and catalytic triad features of OoCBP1 and OoCBP2. (**A**) Pairwise structural alignment of HcCBP1 (green) with OoCBP1 (yellow). (**B**) Structural alignment of HcCBP2 (green) with OoCBP2 (yellow). (**C**) Predicted three-dimensional structure of OoCBP1 highlighting the catalytic triad residues C122, H293, and N313 positioned within the active site cleft. (**D**) Predicted structure of OoCBP2 showing catalytic residues C119, H289, and N313. Structural predictions were generated using AlphaFold 3, and alignments were performed using jFATCAT (Protean 3D, DNASTAR). All structures are shown in ribbon format with catalytic residues labeled for clarity.

OoCBP1 and OoCBP2 comprise 345 and 347 amino acid residues, respectively, with calculated molecular masses of 38.5 kDa and 38.9 kDa. A signal peptide cleavage site was detected between amino acid positions 18 and 19 for OoCBP1 (probability 0.984719) and 15 and 16 for OoCBP2 (probability 0.984757). Sequence alignment with HcCBPs [33] revealed a 53–59% sequence identity, where OoCBP2 and HcCBP2 have the highest sequence identity (59%). Sequence alignment of OoCBPs with *Homo sapiens* cathepsin B (catB), catL, and catK showed that OoCBPs have a greater alignment with catBs but lack one of the two histidine residues in the occluding loop, which is characteristic of the exopeptidase property of human catB (Figure S1). Obviously, the occluding loop sequence is missing in human catL and catK (Figure S1). By comparing to human catB and catL, both proteins appear to possess the conserved Cys, His, and Asn residues that form the canonical catalytic triad of cysteine peptidases [34] (Figure S1). In addition, they have a unique motif (YWLIANSWxxDWGE) attributable to the catB family of proteins for their hemoglobinase activity in blood-feeding helminths [35] and 15 (OoCBP1) or 14 (OoCBP2) conserved Cys residues present in the proteins (Figure S2). The structure–structure-based alignment of HcCBP1 and OoCBP1 had an RMSD alignment score of 2.031 angstroms (Å), and the HcCBP2 and OoCBP2 alignment had an RMSD alignment score of 2.336 Å. An RMSD score between 2 and 3 Å indicates structural similarity (Figure 1A,B). Based on similarity to the UniProt entry P25802, an *O. ostertagi* catB-like cysteine proteinase, the catalytic triad for OoCBP1 is composed of C122, H293 and N313, and that of OoCBP2 is composed of C119, H289 and N313, respectively (Figure 1C,D). The distance between catalytic triad residues ranges from 2.73 to 6.45 Å (Table S1). The sequences of OoCBPs have been deposited in the GenBank database (OoCBP1, accession number PX866999; OoCBP2, accession number PX867000).

### 2.2. OoCBP Expression

**Figure 2:**
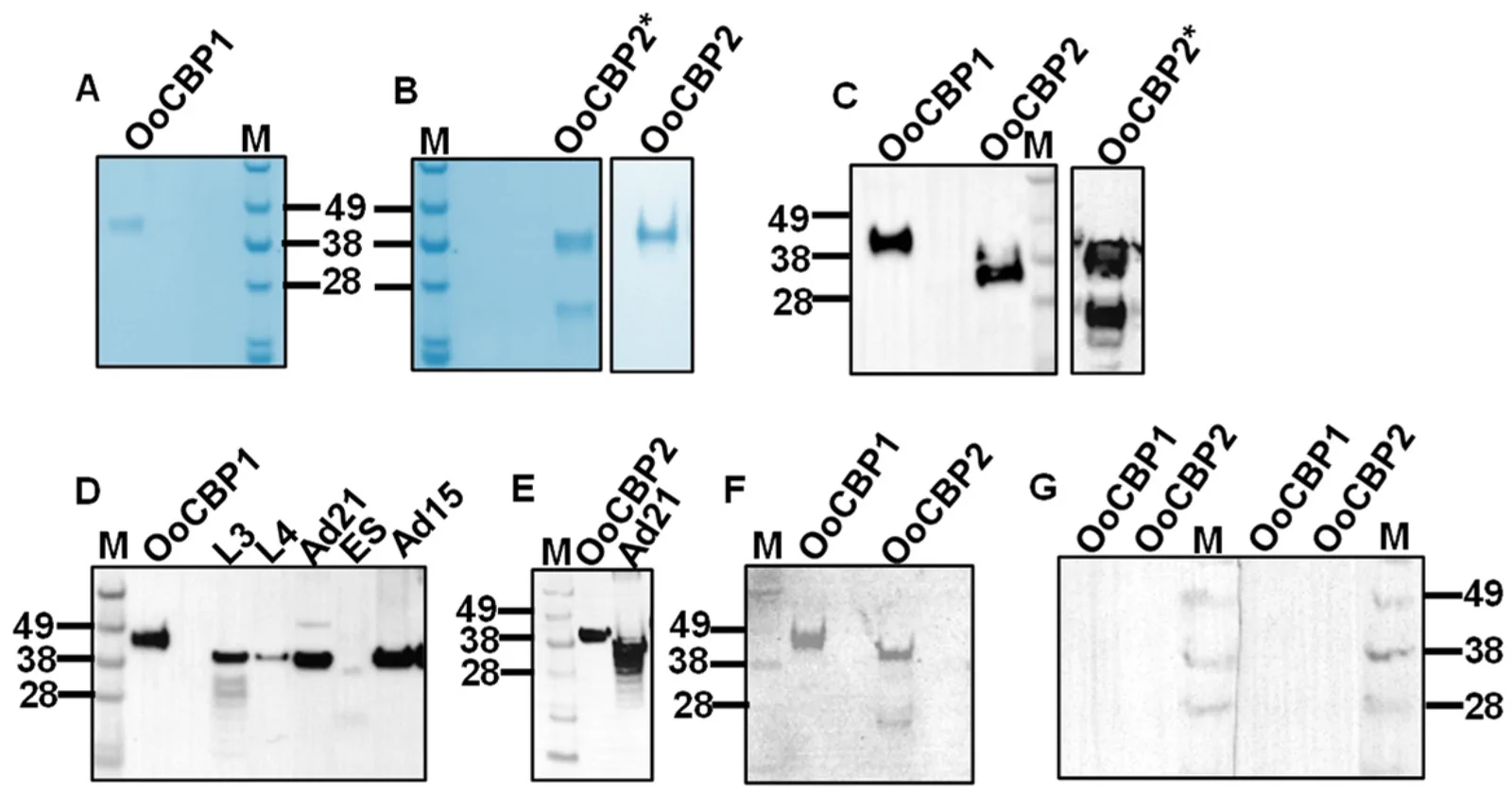
SDS-PAGE and Western blot analyses of native and recombinant *O. ostertagi* cysteine proteases expressed in FreeStyle 293-F cells. (**A**,**B**), OoCBP1 (**A**) and OoCBP2 (**B**), SDS-PAGE with Coomassie blue staining; (**C**), Western blot analysis of OoCBP1 and OoCBP2 with anti-His antibody. (**D**,**E**), Western blot analyses of native OoCBP1 (**D**) and OoCBP2 (**E**) using antisera against OoCBP1 and OoCBP2. (**F**), Western blot analysis of OoCBP1 and OoCBP2 using pooled sera from *O. ostertagi*-infected cattle. (**G**), Western blot analysis of OoCBP1 and OoCBP2 using sera from control cattle. Lane M, protein molecular standards; Ad15 and Ad21, crude extract prepared from day 15 or 21 adult worms, respectively; L3 and L4, crude extract prepared from infective L3 and L4 worms, respectively; ES, concentrated excretory/secretory products of adult worms. OoCBP2, the full-length protein (~39 kDa); OoCBP2*, full-length and autocatalytically processed proteins (~39 and 19 kDa).

OoCBP1 and OoCBP2 were expressed in FreeStyle 293-F cells, purified by affinity chromatography, and purity analyzed by SDS–PAGE (Figure 2A,B). The purified OoCBP1 migrated as a single band at 46 kDa; OoCBP2 migrated as either a single band of 39 kDa or double bands of 39 and 19 kDa (Figure 2A,B). The protein identity was further confirmed by Western blot analysis using the anti-His antibody (Figure 2C).

#### OoCBP Expression Across *O. ostertagi* Life Stages

Western blotting detected OoCBP1 in L3 and L4 larvae and day-15 and -21 adult worms (Figure 2D). OoCBP2 was detected in the adult stage (Figure 2E) but not in the larval stage (Figure S3). To determine if *O. ostertagi*-infected cattle responded to OoCBPs immunologically, Western blotting was employed to detect the serum OoCBP-specific antibodies. Sera pooled from three cattle experimentally infected with *O. ostertagi* detected OoCBP1 as well as OoCBP2 by Western blotting (Figure 2F). Control sera from uninfected cattle failed to detect OoCBPs (Figure 2G).

### 2.3. OoCBP1 and OoCBP2 Are CatL-like Cysteine Proteases Based on Substrate Specificity

**Figure 3:**
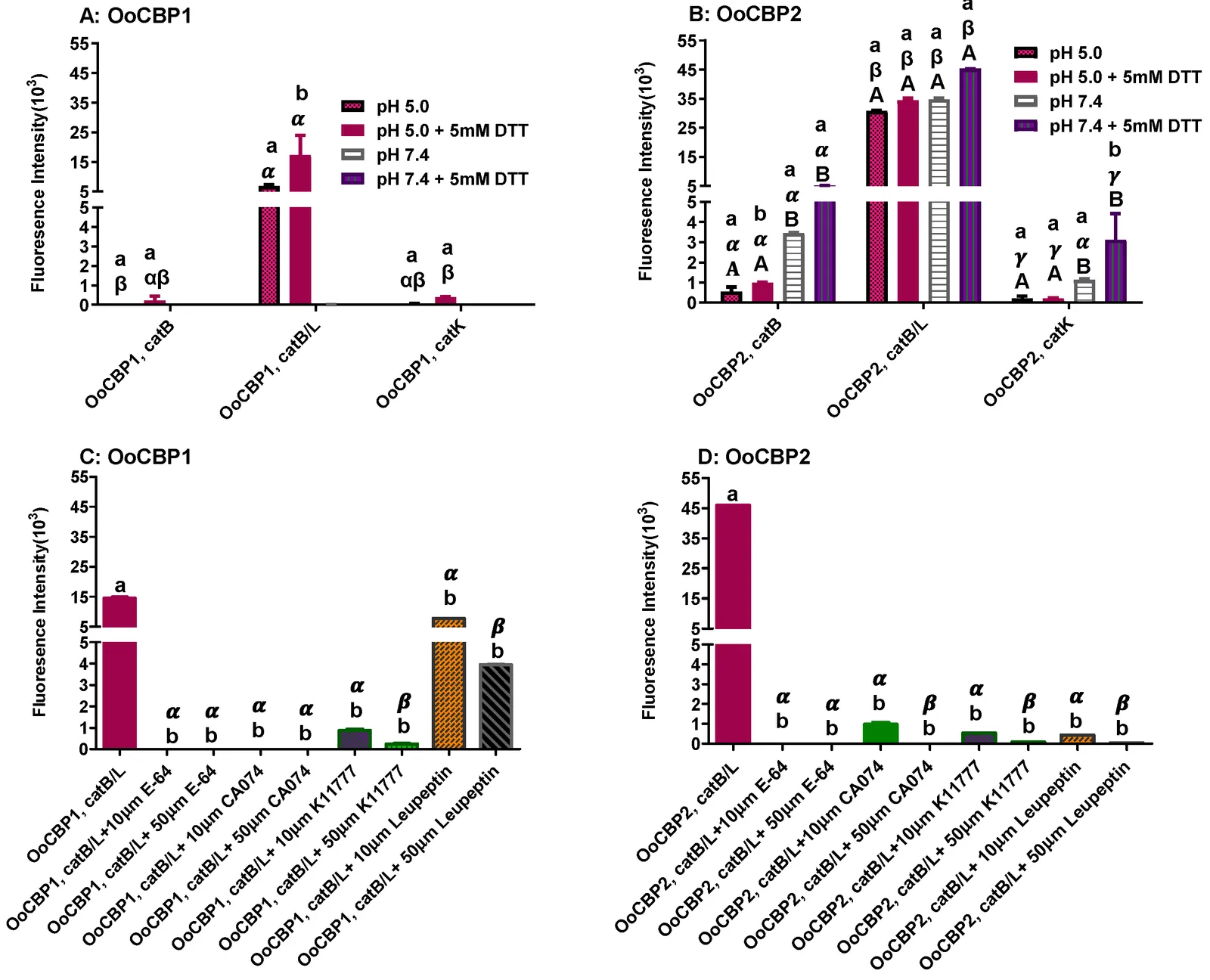
Cathepsin activity of recombinant *O. ostertagi* cysteine proteases (OoCBPs) was assayed using synthetic substrates Z-Arg-Arg-AMC (catB specific), Z-Phe-Arg-AMC (catB/L specific), and Z-Leu-Arg-AMC (catK specific) at 37 °C for 60 min at pH 5.0 (**A**) and 7.4 (**B**) with or without 5 mM DTT. The inhibitory effect of the cysteine protease inhibitor E-64 and catB (CA074), catB/L (K11777), and leupeptin inhibitors on OoCBP1 (**C**) and OoCBP2 (**D**) using the catB/L substrate was determined at pH 5.0 with 5mM DTT (D). For each enzyme (OoCBP1 or OoCBP2), within the same pH or the same pH plus DTT, values with different lowercase Greek letters indicate significant differences (*p* < 0.0001) among substrates (**A**,**B**). For each substrate and within the same pH, values with different lowercase Roman alphabets differ significantly between DTT-treated and untreated samples (**A**,**B**) (*p* < 0.0001). Within a substrate, values with different uppercase Roman alphabets differ significantly (*p* < 0.0001) when different pH or pH plus DTT conditions are compared (**B**). The fluorescence intensity readings were too low or undetectable when substrates were treated with OoCBP1 at pH 7.4 (**A**); thus, the data was not analyzed/shown. For the inhibitor assay results, within the same inhibitor, values with different lowercase Greek letters differ significantly between the 2 concentrations, and values with different lowercase Roman alphabets differ significantly between the untreated control and inhibitor-treated samples (**C**,**D**) (*p* < 0.05). The fluorescence intensity is expressed as mean ± SE.

OoCBP1 and OoCBP2 were predicted as catB-like proteases based on motif analysis and phylogenetic clustering with catB homologues (Figure S2). To assess their substrate specificity, fluorogenic substrate assays were performed using substrates specific to catB (Z-arg-arg-AMC), catK (Z-Leu-arg-AMC) and catB/L (Z-phe-arg-AMC). Low or no activities were detected with OoCBP1 when the catB or catK substrate was used at either pH condition, and catB/L was tested at pH 7.4. OoCBP1 preferentially cleaved the catB/L substrate at pH 5.0, and OoCBP2 was more active with the catB/L substrate at pH 5.0 and pH 7.4 in the presence or absence of DTT when compared to the catB or catK substrate (*p* < 0.0001) (Figure 3A,B). DTT increased (*p* < 0.001) OoCBP1 activity at pH 5.0 on catB/L, whereas DTT elevated the OoCBP2 activity on the catB substrate at pH 5.0 (*p* < 0.05) and the catK substrate at pH 7.4 (*p* < 0.01). OoCBP2 was more active than OoCBP1 on all three substrates under all the conditions tested except catK at pH 5.0 with DTT (Figure 3A,B). Without activation (e.g., at pH 7.4), OoCBP1 had minimum activity on the catB/L substrate (Figure 3A); however, its activity was dramatically increased by in vitro activation at pH 5.0 for 2 h (Figure S4A). Further, a time-dependent increase in enzymatic activity for in vitro-activated OoCBP1 was associated with its gradual downshift of molecular weights (Figure S4B).

Furthermore, we tested the effects of protease inhibitors, including E-64 (cysteine protease inhibitor), catB (CA074), catB/L (K11777), and leupeptin, on OoCBPs using the catB/L substrate and demonstrated that all four inhibitors inhibited (*p* < 0.0001) the OoCBP activity (Figure 3C,D). E-64 abolished the activity of OoCBP1 and OoCBP2, and the catB inhibitor also completely inhibited OoCBP1 activity at both concentrations tested. Both catB/L inhibitor and leupeptin inhibited both enzymes in a dose-dependent manner (*p* < 0.05) (Figure 3C,D).

### 2.4. OoCBPs Degrade Host Protein Substrates

**Figure 4:**
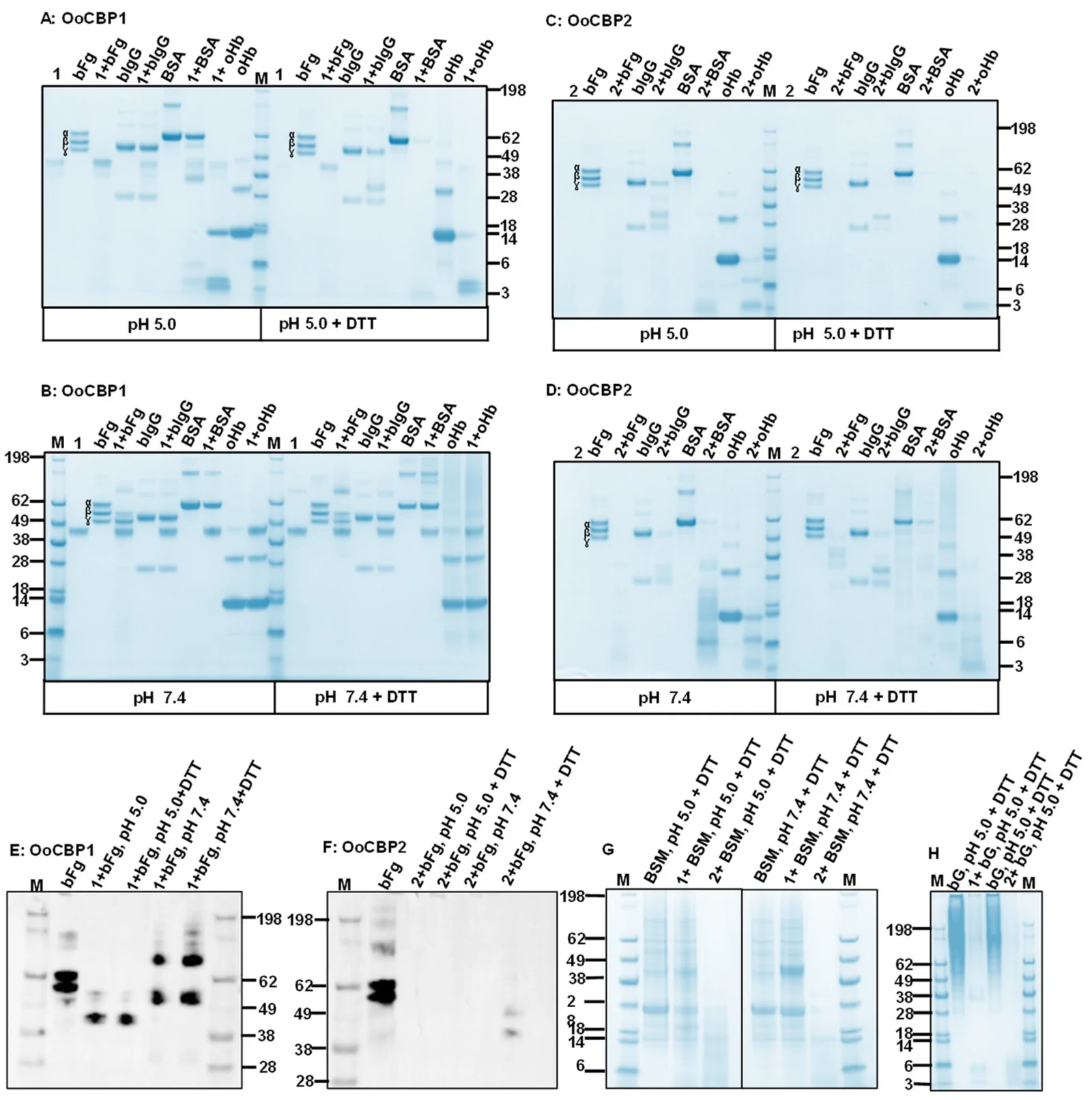
SDS-PAGE analyses of host protein substrates digested by recombinant *O. ostertagi* cysteine proteases, OoCBP1 and OoCBP2, at pH 5.0 or 7.4 in the presence or absence of reducing agent (DTT). Digestion was carried out for 16 h at 37 °C and analyzed by SDS-PAGE followed by Coomassie blue staining (**A**–**D**,**G**,**H**); Western blot analysis of fibrinogen (Fg) degradation (**E**,**F**) by OoCBPs was conducted using anti-bovine Fg antibodies. Lane M, protein molecular standards, OoCBP1 (1), OoCBP2 (2), bovine fibrinogen (bFg), bovine immunoglobulin G (bIgG), bovine serum albumin (BSA), ovine hemoglobin (oHb), bovine submaxillary mucin (BSM), and bovine gelatin (bG).

We evaluated the protease activity of OoCBPs using host protein substrates: ovine hemoglobin (oHb), bovine serum albumin (BSA), bovine immunoglobulin (bIgG), bovine fibrinogen (bFg), bovine gelatin (bG), and bovine submaxillary mucin (BSM) at pH 5.0 and 7.4 and in the presence or absence of DTT (Figure 4A–H, Table 1). OoCBP1 degraded the α (67 kDa), β (57 kDa) and ɣ (47 kDa) chains of bFg at pH 5.0 with or without DTT (Figure 4A). However, at pH 7.4, OoCBP1 only degraded the α band with or without DTT (Figure 4B). In contrast, OoCBP2 completely degraded bFg at both pH 5.0 and 7.4 levels, with or without DTT (Figure 4C,D). OoCBP1 partially degraded the heavy chain (55 kDa) of bIgG only at pH 5.0 in the presence of DTT (Figure 4A). Conversely, OoCBP2 degraded the IgG heavy chain under all conditions tested, whereas it degraded the light chain only at pH 5.0 in the presence of DTT (Figure 4C,D). OoCBP1 degraded BSA (66.5 kDa) most efficiently at pH 5.0 in the presence of DTT (Figure 4A), but not at pH 7.4 with or without DTT (Figure 4B). OoCBP2 completely degraded BSA, including even the oligomeric form of this protein (>192 kDa), at pH 5.0 in the presence or absence of DTT (Figure 4C). OoCBP2 degraded BSA less efficiently at pH 7.4 with or without DTT (Figure 4D). Regarding oHb, its degradation by OoCBP1 was most effective at pH 5.0 in the presence of DTT, hydrolyzing both the monomer and dimer of oHb (α subunit: ~15 kDa, β subunit: ~16 kDa, dimer: 25–35 kDa) into peptide fragments measuring 3–6 kDa (Figure 4A); but no degradation was observed at pH 7.4 with or without DTT (Figure 4B). Conversely, OoCBP2 completely hydrolyzed oHb at pH 5.0 or 7.4 with or without DTT (Figure 4C,D). Degradation of bFg by OoCBP1 or OoCBP2 was further confirmed by Western blotting using an anti-bovine Fg antibody. While OoCBP2 hydrolyzed bFg completely (Figure 4F), OcCBP1 only reduced bFg to a lower molecular fragment at pH 5.0. In addition, OoCBP1 digestion of bFg at pH 7.4 produced a higher-molecular-weight band (~ 84 kDa), as shown in SDS-PAGE (Figure 4B), and a lower-molecular-weight band (41 kDa) (Figure 4E). OoCBP2, but not OoCBP1, was able to degrade BSM at both pH 5.0 and pH 7.4 (Figure 4G). Both OoCBP1 and OoCBP2 demonstrated the ability to degrade bG at pH 5.0 in the presence of DTT (Figure 4H).

### 2.5. Temporal Kinetics of Fg Degradation by OoCBPs and Their Inhibition by Cysteine Protease Inhibitors

**Figure 5:**
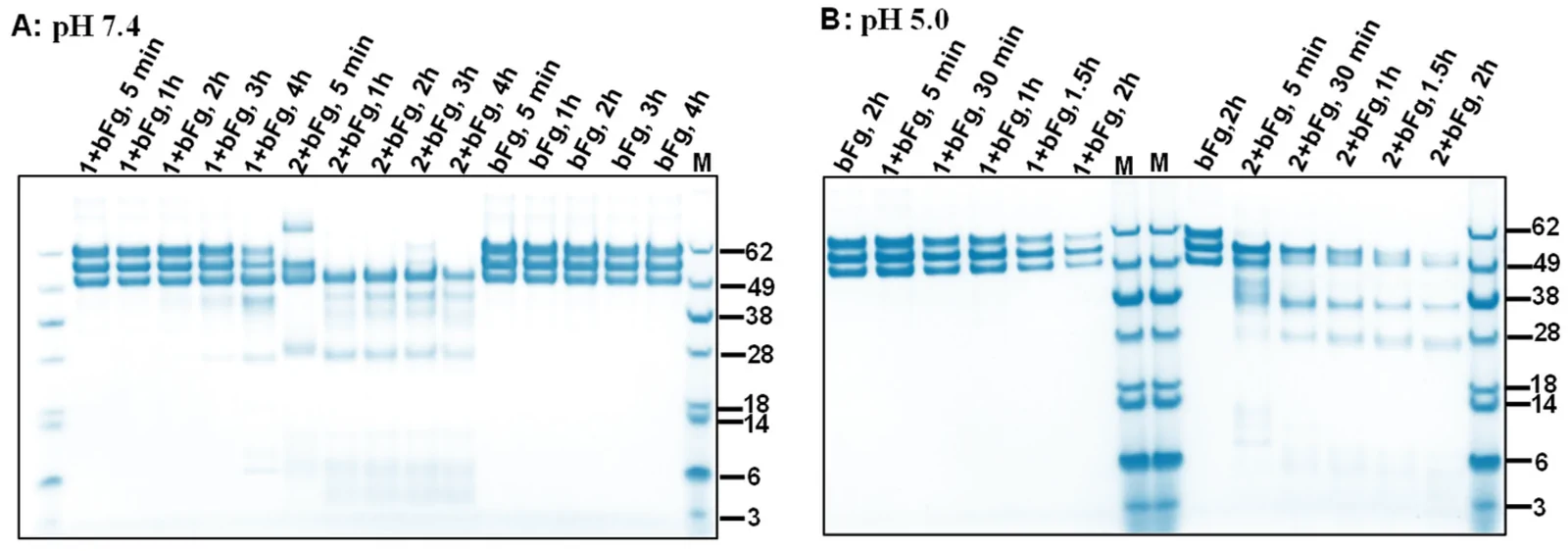
SDS-PAGE analysis of time-dependent degradation of bovine fibrinogen (bFg) by OoCBP1 and OoCBP2 at 37 °C in PBS at pH 7.4 (**A**) and in 100 mM sodium acetate buffer containing 2 mM DTT and 2 mM EDTA, pH 5.0 (**B**). Proteins were separated under reducing conditions using SDS-PAGE followed by Coomassie Blue staining. (**A**) Aliquots were collected from the digestion for analysis at 5 min, 1 h, 2 h, 3 h, or 4 h. (**B**) Aliquots were collected from the digestion for analysis at 5 min, 30 min, 1 h, 1.5 h, or 2 h. Lane M, protein molecular standards; 1, OoCBP1; 2, OoCBP2; bFg, bovine fibrinogen.

**Figure 6:**
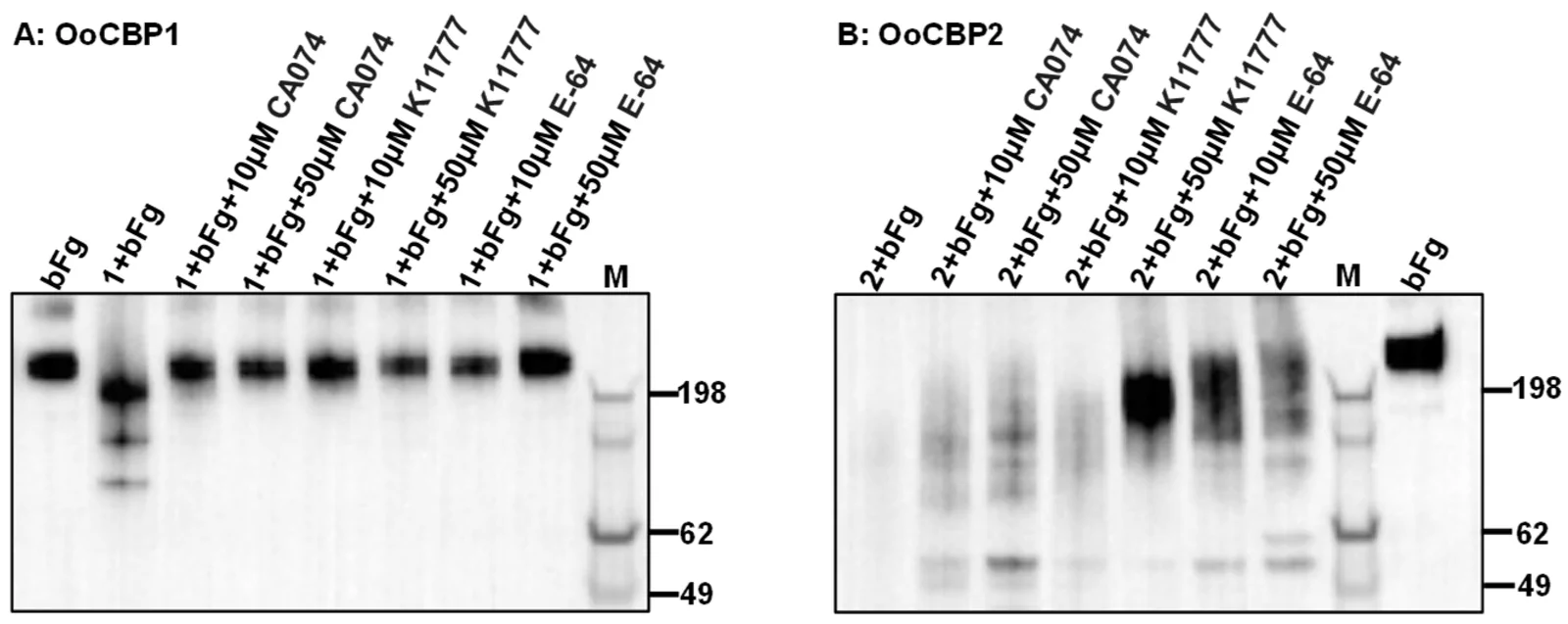
Western blot analysis of bFg degradation by OoCBP1 (**A**) and OoCBP2 (**B**) in the presence of protease inhibitors, including cysteine protease inhibitor (E-64), catB inhibitor (CA074), and catB/L inhibitor (K11777). OoCBPs were preincubated with 10 μM and 50 μM of each of the inhibitors for 5 min before adding bFg. The mixture was incubated for 16 h at 37 °C at pH 7.4 and analyzed by using anti-bovine Fg antibodies in non-reducing conditions. A and B. Lane M, protein molecular standards, OoCBP1 (1), OoCBP2 (2), bovine fibrinogen (bFg).

The degradation of bFg by OoCBP1 at pH 7.4 started to occur at 3 h in incubation (Figure 5A); however, at pH 5.0, degradation was evident at 1.5–2 h (Figure 5B). In contrast, bFg degradation by OoCBP2 began as early as 5 min in incubation with complete degradation of the alpha chain under both pH conditions and resulted in the formation of a higher-molecular-weight band (~94 kDa) at pH 7.4 (Figure 5A,B). OoCBP2 completely degraded both the alpha and beta chains of bFg at pH 7.4 when incubated for 2–4 h, and at pH 5.0, degraded the alpha chain but only gradually degraded the beta chain between 5 min and 2 h (Figure 5A,B). The ability of OoCBP1 to degrade bFg was effectively inhibited by E-64 and catB- and catB/L-specific inhibitors (Figure 6A and Figure S5A). Out of the three cysteine protease inhibitors used, the catB inhibitor had minimal effect, but E-64 had a consistent and potent inhibitory effect at 10 or 50 µm concentrations on OoCBP2 activity. The catB/L-specific inhibitor was most effective only at 50 µm concentration (Figure 6B and Figure S5B).

### 2.6. Effect of Recombinant OoCBPs on Clotting Time

**Figure 7:**
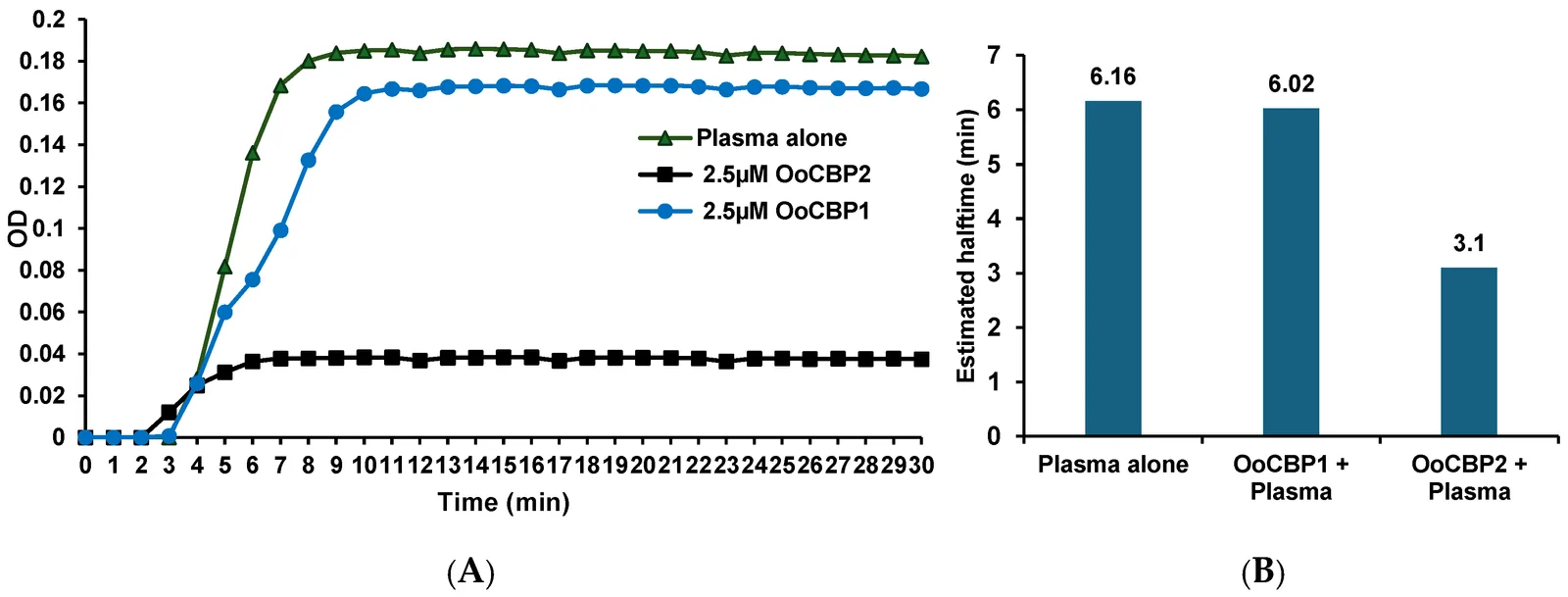
Effects of recombinant *O. ostertagi* cysteine proteases (OoCBPs) on plasma clotting by recalcification assay (RCA). (**A**) RCA using human reference plasma to evaluate the impact of OoCBPs on coagulation. The plates were read at 20 s intervals over a 30 min period using a spectrophotometer set to 37 °C. All assays were repeated 3 times in duplicate. (**B**) Effect of OoCBPs on the halftime of plasma clotting determined by RCA. Halftime is defined as the plasma clotting time required to reach half-maximum absorbance.

In the RCT assay, OoCBP1 and OoCBP2 were evaluated at 2.5 μM; the estimated clotting halftime was 6.16 min (95% confidence interval (CI): 6.02–6.31 min) for untreated human plasma and 6.02 min (95% CI: 5.61–6.43 min) and 3.10 min (95% CI: 2.82–3.38 min) for plasma treated with OoCBP1 and OoCBP2, respectively. The data indicate that the plasma clotting halftime was significantly reduced by OoCBP2 but not OoCBP1. Analysis of clot density at 30 min showed that OoCBP2 reduced the clot density compared with either the plasma-alone control or OoCBP1-treated plasma (*p* < 0.0001). OoCBP1 treatment did not differ from the plasma-alone control in clot density (*p* > 0.05) (Figure 7A).

In the FPA using OoCBPs without in vitro activation, bFg treated with OoCBP1 exhibited estimated clotting halftimes that did not differ from those of untreated bFg following 15 min incubation time (11.43 min with 95% CI of 11.33–11.52 min vs. 11.38 min with 95% CI of 11.19–11.57 min). In contrast, bFg treated with OoCBP1 for 30 min displayed an increased clotting halftime when compared to untreated bFg (12.18 min with 95% CI of 12.09–12.27 min vs. 11.53 min with 95% CI of 11.31–11.74 min).

**Figure 8:**
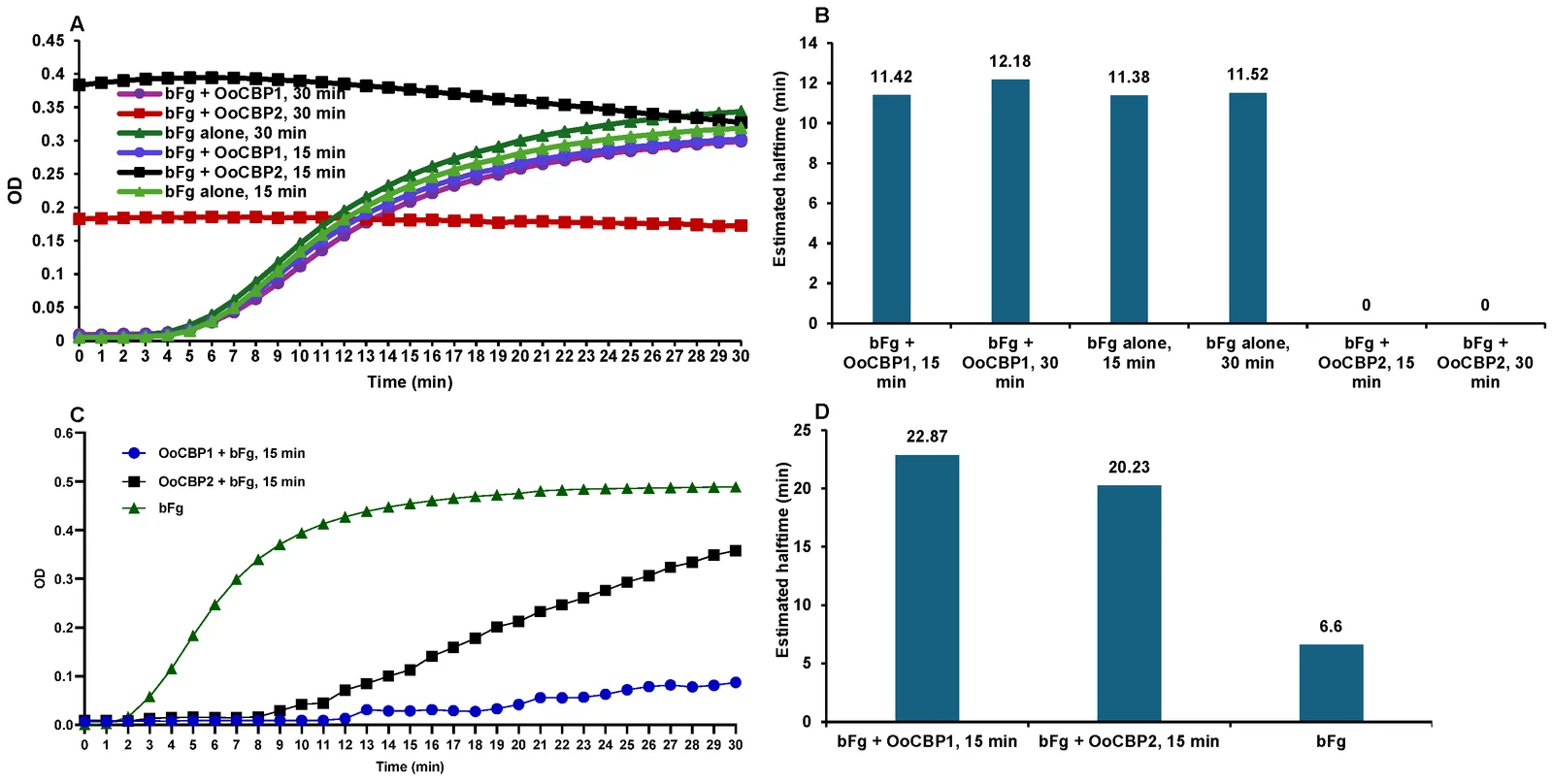
The effect of recombinant *O. ostertagi* cysteine proteases (OoCBPs) on clotting by using the fibrin polymerization assay (FPA) using bovine fibrinogen. (**A**) FPA performed in PBS, pH 7.4. (**B**) Effect of OoCBPs on the halftime of fibrin polymerization determined by FPA in PBS at pH 7.4. (**C**) FPA performed under identical conditions after pre-exposing OoCBPs to pH 5.0 and readjusting to PBS, pH 7.4. (**D**) Effect of OoCBPs on the halftime of fibrin polymerization determined by FPA performed under identical conditions after pre-exposing OoCBPs to pH 5.0 and readjusting to PBS, pH 7.4. We read the plates at 20 s intervals over a 30 min period using a spectrophotometer set at 37 °C. Halftime is defined as the clotting time required to reach a half-maximum absorbance value.

The bFg pretreated with OoCBP2 for 15 or 30 min initiated clot formation immediately following thrombin addition, resulting in significantly different (*p* < 0.0001) clot density between the two incubation times (Figure 8A). Clot density of bFg subjected to 15 min preincubation with OoCBP2 was not significantly different (*p* > 0.05) from that of the control; however, clot density with 30 min preincubation was significantly reduced (*p* < 0.0001). Whereas OoCBP1-treated fibrinogen exhibited lower clot density than the control and OoCBP2. At 30 min, both OoCBP1- and OoCBP2-treated fibrinogen showed clot density significantly lower than in the untreated control (*p* < 0.05), with no significant difference between the two enzyme treatments (Figure 8B). However, treating bFg with OoCBP2 for 30 min resulted in lower clot density. By evaluating the activities of OoCBPs after in vitro activation using the FPA, we found that untreated bFg had a halftime of 6.61 min (95% CI: 6.50–6.72 min), and bFg treated with OoCBP1 or OoCBP2 showed estimated halftimes of 22.87 min (95% CI: 13.05–32.69 min) and 20.23 min (95% CI: 16.90–23.56 min), respectively. Both OoCBP1 and OoCBP2 treatments resulted in reduced clot density compared with the control (*p* < 0.0001) (Figure 8C,D).

## 3. Discussion

In this study, we identified two novel cysteine proteases from *O. ostertagi*, OoCBP1 and OoCBP2. These are orthologs of cathepsin B-like proteases 1 and 2 (HcCBP1 and HcCBP2) from *H. contortus* [32]. OoCBPs demonstrate greater alignment with human catB, but with the absence of the occluding loop [36]. This occluding loop prevents cystatin-like protein inhibitors from binding to catB, in contrast to their interaction with papain [37]. Therefore, OoCBPs are unique; the inhibitors designed to target these parasite proteins may represent a distinct class of chemotherapeutic agents for combating the parasite; however, their potential cross-reactivity with the host cathepsins remains to be determined experimentally. Structural analysis of these enzymes indicates the presence of a catalytic triad typical of cathepsins. This structural validation strengthens the functional relevance of the biochemical properties demonstrated in this study. As OoCBPs contain a signal peptide, they are presumably secreted. These proteases show expression across *O. ostertagi* life stages, from infective L3 to adults, suggesting a stage-specific, functional role in the establishment of infection within the host [38]. Indeed, we detected OoCBP-specific antibodies in *Ostertagia*-infected cattle, suggesting that native OoCBPs released from the parasite were immunogenic to the host.

We produced and characterized the mammalian cell-expressed recombinant OoCBPs both biochemically and functionally to assess their ability to simulate the native counterparts. Recombinant OoCBP1 migrated at a higher molecular mass than predicted from its amino acid sequence. This difference may be attributable to post-translational modifications acquired during expression in FreeStyle 293-F cells, and it is consistent with the observation that the *H. contortus* ortholog of OoCBP1 also exhibited an increased apparent molecular weight when expressed in mammalian cells compared with the corresponding recombinant protein produced in a bacterial expression system [33]. Recombinant OoCBP2 was self-activated, possibly by slitting off the pro-domain under the in vitro culture conditions during production, as shown with catB from *S. mansoni*, which is activated as produced [39]. In practice, this process negates the necessity for additional in vitro activation. OoCBP2 hydrolyzes catB/L substrates as well as protein substrates at pH 7.4 without additional activation; in contrast to OoCBP1, which exhibits greater activity at pH 5.0, necessitating a low pH environment for autoactivation. We show that OoCBPs are most effective in hydrolyzing the catB/L substrate when compared to the catB and catK substrates, suggesting that OoCBPs are catB/L-like proteases. Likewise, similar catB-like proteins were described in parasitic nematodes, which feature the catB characteristics but with a catL substrate specificity [33,40,41]. However, all protease inhibitors used in this study significantly inhibited both OoCBP1 and OoCBP2 [42]. In the presence of a reducing agent, the catB inhibitor became more potent and cross-inhibited both OoCBP1 and OoCBP2. As shown previously, reducing conditions rendered one of the catB-specific inhibitors increased potency against human catL. It is likely that reducing agents alter the non-catalytic cysteine bonds, inducing subtle conformational changes that open the binding pocket and increase inhibitor accessibility [43].

The present findings indicate that both OoCBPs exhibit broad proteolytic activity against major host proteins, suggesting that OoCBPs may play an essential role in protein digestion and nutrient acquisition within both the acidic and neutral compartments, such as the parasite’s gut and/or the host–parasite interface [1]. During the adult phase, when nutritional requirements increase, which coincides with mucosal bleeding caused by adult worms exiting from the gastric glands, results in blood components being ingested [44]. The capacity of OoCBPs to degrade host IgG, with apparent specificity for the heavy chain, further supports their possible role in immune evasion strategies, although this remains to be demonstrated in vivo. Similar IgG hydrolysis has been described in several GI nematodes, supporting the notion that antibody degradation may represent a conserved mechanism to counter host immune defenses [45]. The observed hemoglobinolytic activity of OoCBPs, likely conferred by a hemoglobinase motif, indicates an additional function in heme acquisition, an essential process given that *O. ostertagi* lacks the de novo heme biosynthetic pathway [46]. Moreover, during clinical ostertagiosis, one of the major pathophysiological manifestations is impaired GI function due to the loss of parietal and chief cells. This leads to a change in pH from 2.0–3.0 to 5.0–6.0 or even higher. Additionally, the microenvironment around the parasite may differ significantly from that in the lumen due to mucus secretion, immune cell responses, and leakage of plasma proteins [47]. Under these conditions, the ability of OoCBPs to remain active over a pH range (5.0 to 7.4) may offer a significant adaptive advantage, ensuring consistent protein degradation and nutrient absorption. Given their multifunctional roles in nutrient utilization and immune modulation, OoCBPs warrant further investigation as potential targets for vaccine or therapeutic development.

Mucins, the major proteinaceous constituent of mucus, are heavily glycosylated proteins in the GI tract that serve as a formidable physical barrier to microbial invasion [48,49]. OoCBP2 degrades mucins as shown in our study, suggesting its potential role in facilitating penetration through the mucous layer when burrowing into or exiting from the gastric glands [50]. Adult *O. ostertagi* worms are found on the surface of the abomasal mucosa, while the L4 reside within the gastric glands, where the parasite is closely associated with the abomasal mucosa. The secreted proteases are likely critical for establishing persistence. OoCBP1 and OoCBP2 degrade bovine gelatin (denatured collagen) at acidic pH, which simulates the abomasal environment where parasite cysteine proteases may play a role in molting by breaking down the collagen-rich cuticle [51]. Our results are consistent with studies involving cysteine proteases in helminths that degrade both gelatin and mucin [52,53].

It is intriguing that, upon digestion by OoCBP2, a transient higher-molecular-weight Fg product and a lower-molecular-weight fragment were generated. The identities of these bands could not be confirmed from our data. However, their approximate migration closely resembles that of cleavage products previously described for papain-treated Fg, in which a higher-molecular-weight band corresponding to ɣ–ɣ dimers (94 kDa) and an ~25-kDa α-chain fragment appear in the lower-molecular-weight range. Although our observations are consistent with these prior patterns, any direct correspondence remains tentative and should be interpreted with caution.

The Fg degradation by OoCBPs supports a potential anticoagulant function of these proteases, suggesting that maintaining blood fluidity is essential for continued feeding by the worms. The two proteases may play unique roles in this process. At the physiological pH of host blood, OoCBP1 has no effect on coagulation, whereas OoCBP2 produces a distinct clot characterized by drastically reduced density. We understand that human reference plasma was used for bovine coagulation in our study. This is because the use of human reference plasma will allow for data comparisons across different studies [54,55]. Given that there is a potential interspecies difference in coagulation, we used bovine Fg for some of our coagulation tests. While the present findings support the anticoagulation activity of OoCBP1 and OoCBP2 under in vitro conditions, future studies comparing bovine and human plasma, as well as in vivo investigations, will be valuable to further define their physiological relevance in ruminants.

Both OoCBPs behave differently with or without in vitro activation. Without in vitro activation, OoCBP1 had no effect on clotting, regardless of whether human reference plasma or purified Fg was used; however, OoCBP2 mediated immediate Fg clotting. The immediate Fg clotting mediated by OoCBP2 may be because of the formation of γ chain dimers, which can result in higher clot density [56]. This suggests that OoCBP2 potentiates thrombin’s action by processing Fg to promote rapid polymerization. This is consistent with the papain effect on human Fg, where a low-density clot was induced [57]. Yet, when OoCBPs were subjected to in vitro activation, both proteins were highly activated to delay Fg clotting, resulting in reduced clot density. Our data also suggest that OoCBP2 may possess two discrete biological functions regulating coagulation: one is to promote fibrin polymerization when it is autoactivated but not activated by the acidic condition, and the other is to break down Fg when it is fully activated by acid. We believe that this functional switch may reflect pH-dependent differences in Fg processing, where a neutral pH environment may help expose thrombin-sensitive regions, allowing for rapid clotting, whereas acidic pH promotes the Fg α chain degradation that likely slows protofibril assembly, but without completely preventing clot formation [58]. The data suggest that both proteases are key to protein degradation and anticoagulation; however, their precise biological functions within the parasite’s gut remain to be determined [59].

**Figure 9:**
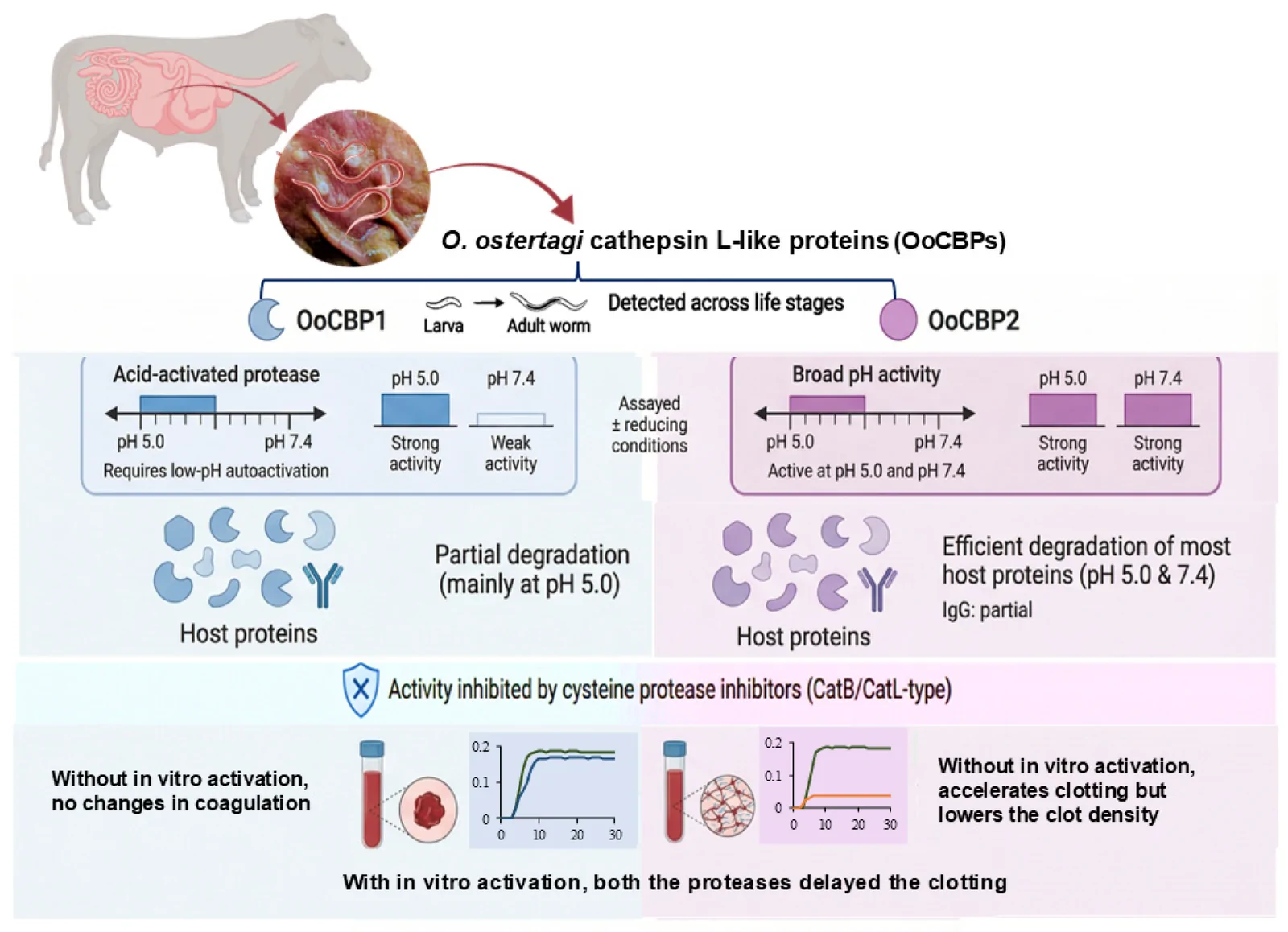
Schematic representation of the role of *O. ostertagi* cysteine proteases (OoCBPs) in facilitating parasite survival in the host abomasum (created in BioRender. Mekhemadhom Rajendrakumar, A. (2026) https://core.local.biorender.dev/api/short-link/jfvvsyv, accessed on 9 October 2026).

In summary, this study has identified two novel cysteine proteases from *O. ostertagi*, OoCBP1 and OoCBP2, which are functionally active catL-like enzymes. Their pH-dependent activation and broad host protein substrate specificity illustrate their potential physiological relevance across various parasitic life stages, suggesting possible roles in host–parasite interactions, nutrient acquisition, regulation of coagulation, and immune evasion (Figure 9). Interrupting these processes could dramatically hinder the parasite’s ability to survive within the host. Thus, OoCBP1 and OoCBP2 may stand as promising molecular targets for developing vaccines and therapeutic interventions to control bovine ostertagiosis and reduce reliance on anthelmintics.

## 4. Materials and Methods

### 4.1. Bioinformatics Analysis of Cysteine Proteases (OoCBP1 and OoCBP2) of O. ostertagi

The sequences of cysteine proteases (CPs) from *H. contortus*, specifically HcCBP1 (accession number CDJ83387.1) and HcCBP2 (accession number CDJ87123.1), were used to identify OoCBP1 and OoCBP2 from the *O. ostertagi* transcriptome using tblastn with the open source Galaxy tool [60]. The Clustal W 2.1 and Clustal Omega1.2.4 were utilized for sequence alignment with default parameters for comparing OoCBP sequences with *Homo sapiens* cathepsin B (catB), catL and catK as well as with HcCBPs [61] (https://www.ebi.ac.uk/jdispatcher/msa/clustalo) (accessed on 9 October 2025). The signal peptide sequences were predicted by the SignalP 6.0 server (https://services.healthtech.dtu.dk/services/SignalP-6.0/) (accessed on 16 May 2024) with default parameters [62].

The structures of OoCBP1, OoCBP2, HcCBP1 and HcCBP2 were predicted using AlphaFold 3 [63]. Pairwise structure–structure alignments were performed for HcCBP1 and OoCBP1 as well as HcCBP2 and OoCBP2 using the jFATCAT in Protean 3D of DNAstar version 18.1.1 (DNASTAR, Inc., Madison, WI, USA) [64]. The catalytic-site prediction was based on sequence similarity. OoCBP1 and OoCBP2 protein sequences were searched against the Swiss Prot database (12 March 2026) using BLASTP (version 2.15.0). The active sites of the top homologs were then retrieved. The top homologs (E-value Cutoff ≤ 1 × 10^−5^) were aligned using the NEEDLE program EMBOSS:6.6.0.0 [65], which utilizes a Needleman–Wunsch algorithm [66] for global pairwise sequence alignment, and, based on the resulting alignment, the query positions and the active-site residues were identified. The graphs of active site structures were created by using Protean 3D of DNAstar version 18.1.1 (DNASTAR, Inc., Madison, WI, USA). A schematic overview summarizing the complete methodological workflow employed in this study is provided in Figure S6.

### 4.2. Recombinant O. ostertagi OoCBP Production

Gene cloning, synthesis, and protein expression were carried out as described previously [33]. In brief, the *OoCBP1* and *OoCBP2* genes were cloned into the pcDNA3.1 (Invitrogen, Waltham, MA, USA) vector for expression in FreeStyle 293-F cells (Invitrogen, Waltham, MA, USA). To enhance secretion and improve the expression of properly folded and post-translationally modified recombinant proteins, the original signal peptide was replaced with the CD5 signal sequence. Additionally, a His tag was added to the C-terminus of the protein to facilitate detection and purification. The recombinant construct was transformed into XL2-Blue ultra-competent cells (Agilent, Santa Clara, CA, USA), and the plasmid DNA was purified utilizing the Qiagen Maxi Prep kit (Qiagen, Germantown, MD, USA). For transfection, 1 µg of plasmid DNA and 3 µg of PEI Max 40,000 (Polysciences, Warrington, PA, USA) were mixed and incubated at room temperature for 10 min, then used to transfect 1 million cells with 98–99% viability. The cells were incubated at 37 °C with 8.0% CO_2_ on a shaker at 135 rpm. Aliquots of the culture supernatants were collected every 24 h to evaluate cell viability and protein expression by SDS-PAGE and Western blotting using a monoclonal anti-His antibody (Invitrogen, Waltham, MA, USA). After an incubation period of 5 to 7 days, the culture supernatant was harvested by centrifuging the cell suspension at 3500 RCF for 30 min at 4 °C.

### 4.3. Purification of OoCBPs

OoCBPs were purified by immobilized metal ion affinity chromatography (IMAC) utilizing Ni-NTA agarose (Qiagen, Germantown, MD, USA). The harvested cell supernatants were filtered through a 0.45 µM filter (Thermo Fisher Scientific, Waltham, MA, USA) and concentrated using a 3 kDa MWCO Centricon Plus-70 centrifugal filter (MilliporeSigma, Burlington, MA, USA). The concentrated supernatant was purified by affinity chromatography as described [33]. The purified proteins were analyzed for purity using a 4–12% Bis-Tris gradient gel (Invitrogen, Waltham, MA, USA) and stained with the Simply Blue Safe Stain (Thermo Fisher Scientific, Waltham, MA, USA). Protein concentrations were quantified using a NanoDrop 2000 spectrophotometer (Thermo Fisher Scientific, Waltham, MA, USA). The purified proteins were stored at −80 °C until use.

### 4.4. Polyclonal Antibody Production

Six to eight-week-old female C57BL/6 mice (*n* = 3 for each protein; *n* = 3 for naïve controls) were intramuscularly immunized at weeks 1, 4 and 5 with the purified OoCBP1 or OoCBP2 formulated in the adjuvant CpG (InvivoGen, San Diego, CA, USA) plus Imject Alum (Thermo Fisher Scientific, Waltham, MA, USA). Blood samples were collected to monitor antibody production by ELISA. Pooled sera from 6- to 9-month-old *O. ostertagi*-infected (*n* = 3) and uninfected Holstein steers (*n* = 3) of a previous experiment were used to test for the presence of infection-elicited anti-OoCBP antibodies. The animal use and care protocol was approved by the Institutional Animal Use and Care Committees (IACUC) (protocol: R-MAR-23-13; AUP# 14-012).

### 4.5. SDS-PAGE and Western Blot Analysis

The proteins were separated using 4–12% Bis-Tris NuPAGE (Invitrogen, Waltham, MA, USA) under reducing conditions, followed by Coomassie blue staining with the Simply Blue Safe Stain (Thermo Fisher Scientific, Waltham, MA, USA). For Western blotting, the proteins were transferred onto a polyvinylidene difluoride (PVDF) membrane (MilliporeSigma, Burlington, MA, USA), blocked using 3% skim milk in PBS with 0.1% Tween 20, and probed with a primary antibody followed by detection with an appropriate HRP-conjugated secondary antibody. The blot was developed using the Super Signal West Dura Extended Duration horseradish peroxidase substrate (Thermo Scientific, Waltham, MA, USA) and the image was acquired using the ChemiDoc System (Bio-Rad, Hercules, CA, USA).

### 4.6. Stage-Specific Expression of OoCBP1 and OoCBP2 in O. ostertagi

Infective L3 and L4 (day 9) and days 15 and 21 adult *O. ostertagi* were processed for crude worm protein preparation using a pulverizing mill in liquid nitrogen (SPEX SamplePrep Freezer/Mills 6875, Metuchen, NJ, USA). The worm powder was dissolved in Ca^++^- and Mg^++^-free PBS (Thermo Fisher Scientific, Waltham, MA, USA), centrifuged, and the supernatant was collected for further analysis. The ES products of *O. ostertagi* were obtained by culturing the adult worms; the resultant supernatant was concentrated for analysis. Protein concentrations were quantified using the Bradford protein assay kit (Bio-Rad, Hercules, CA, USA). The presence of anti-OoCBPs antibodies in sera of *O. ostertagi*-infected cattle was detected using the recombinant OoCBPs.

### 4.7. Protease (Cathepsin) Activity and Inhibition Assays

Protease activity was measured using fluorogenic aminomethylcoumarin-coupled (AMC) peptide substrates, including Z-arg-arg-AMC (catB), Z-phe-arg-AMC (catB/L) (MilliporeSigma, Burlington, MA, USA) and Z-Leu-arg-AMC (catK) (Bachem Americas, Torrance, CA, USA). The assay was conducted in a microtiter plate format using a fluorimeter (FluoStar Optima microplate reader, BMG Labtech, Ortenberg, Germany) [33]. In brief, reactions (200 µL) were prepared in either 20 mM MES, pH 5.0, or 1× PBS, pH 7.4, with or without 5 mM DTT. OoCBPs (0.25 µM) were preincubated in the respective buffer for 15 min at 37 °C, after which substrate was added to 50 µM to initiate the reaction. Assays were maintained at 37 °C, and fluorescence was recorded at 15 min intervals for 1 h (Ex 355 nm/Em 460 nm).

Inhibition of the protease activity of both OoCBP1 and OoCBP2 was tested at pH 5.0 (20 mM MES buffer with 5 mM DTT) with 50 μM catB/L substrate using inhibitors at 10 μM and 50 μM concentrations, including L-trans-epoxysuccinyl-leucyl-amido (4-guanidino) butane (E-64), an irreversible broad spectrum Clan CA cysteine protease inhibitor; N-methylpiperazine-ureaphenylalanyl-homophenylalanyl-vinylsulfone-benzene (K11777), an irreversible inhibitor that targets catB/L-like cysteine proteases; N (L-trans-propylcaramoyloxirane-2-carbonyl)-L-isoleucyl-proline (CA074), an inhibitor of catB-like cysteine proteases [67]; and leupeptin, the naturally occurring, reversible inhibitor against a broad spectrum of proteases, including cat (B, H, and L). These inhibitors and OoCBPs were preincubated for at least 5 min before adding catB/L-specific substrate. In addition, the proteolytic activity of in vitro-activated OoCBP1 in 100 mM sodium acetate with 2 mM DTT and 2 mM EDTA at pH 5.0 for up to 2 h was determined using a substrate specific to catB/L. The integrity of in vitro-activated OoCBP1 was analyzed by SDS-PAGE. Three independent experiments (*n* = 3) were conducted with 2–3 technical replicates for cathepsin and cathepsin inhibition assays.

### 4.8. Degradation of Host Protein Substrates by OoCBPs

The ability of OoCBPs to hydrolyze ovine hemoglobin (oHb), bovine serum albumin (BSA), bovine immunoglobulin G (bIgG), bovine fibrinogen (bFg), bovine gelatin (bG), and bovine submaxillary mucin (BSM) was tested. All protein substrates employed in this experiment were procured from MilliporeSigma (Burlington, MA, USA) except BSM, which was purchased from MP Biomedicals (Fountain Parkway, Solon, OH, USA). The substrates at a final concentration of 2 mg/mL were incubated overnight at 37 °C with OoCBPs (1 μg) in a total volume of 30 μL, using either 20 mM MES buffer, pH 5.0, or PBS buffer, pH 7.4, with or without 5 mM DTT. Control reactions contained substrates without OoCBPs. Aliquots (20 μL) of the resulting hydrolysates were mixed with LDS-PAGE sample buffer, separated by SDS–PAGE, and subsequently stained with Coomassie blue. Degradation of bFg was analyzed by Western blotting using anti-bovine Fg antibody (Abbexa, Cambridge, UK). The protein degradation assays were repeated in three independent experiments, and the representative results are presented.

### 4.9. Temporal Kinetics of bFg Degradation by OoCBPs and Effect of Cysteine Protease Inhibitors on bFg Degradation

The temporal kinetics of bFg degradation by OoCBPs were analyzed by co-incubating bFg and OoCBPs in PBS at pH 7.4 or 100 mM sodium acetate with 2 mM DTT and 2 mM EDTA at pH 5.0. Aliquots were collected into LDS-PAGE sample buffer at various time points to monitor digestion by SDS-PAGE. Following the electrophoresis, the degradation products were visualized by Coomassie blue staining. In addition, to determine the enzymatic specificity, 10 or 50µM of the cysteine protease inhibitors, including E-64 and those specific for catB and catB/L, were preincubated with OoCBPs for 5 min before adding bFg. The reaction was carried out using PBS at pH 7.4 or MES buffer at pH 5.0 at 37 °C for 16 h.

### 4.10. Regulation of Blood Coagulation by OoCBPs

The effects of OoCBPs on blood clotting were assessed using a fibrin polymerization assay (FPA) and a recalcification assay (RCT) in 96-well microplates [68]. The RCT assay was performed as described elsewhere with modifications [33,69]. Briefly, 50 μL of the universal coagulation reference human plasma (UCRP) (DiaPharma, Louisville, KY, USA) was preincubated with or without 2.5 μM of OoCBP1 or OoCBP2 in 10 mM HEPES buffer containing 0.15 M NaCl, pH 7.4, at 37 °C for 15 min. The coagulation was then activated by the addition of 20 μL of 50 mM CaCl_2_. Clot formation was monitored at 650 nm at 20 s intervals for 30 min using a spectrophotometer at 37 °C (SpectraMax 190, Molecular Devices, San Jose, CA, USA). The RCT was performed in three independent experiments (*n* = 3) with 2 technical replicates.

In FPA, thrombin cleaves bFg to produce fibrin monomers, which then polymerize to form a fibrin clot. Anticoagulants that interfere with fibrin polymerization will delay or prevent clot formation. Specifically, to assess the effect of OoCBPs on clotting, 40 μL of bFg, 6.25 mg/mL, was preincubated with OoCBP1 and OoCBP2 (6 µg/10 µL) in PBS at pH 7.4 at 37 °C for 15 or 30 min. Clotting was initiated by adding 50 μL of the thrombin (MP Biomedicals, Solon, OH, USA) solution (5 units/mL in 50 mM HEPES containing 140 mM NaCl and 10 mM CaCl_2_, pH 7.4). The polymerization reaction was monitored using a spectrophotometer at 350 nm at 20 s intervals over a 30 min period at 37 °C. Additionally, we conducted FPA using in vitro-activated OoCBP1 and OoCBP2 in 100 mM sodium acetate with 2 mM DTT at pH 5.0. As this assay uses purified bFg, this method allowed for standardization by avoiding the complexities of human reference plasma used in the RCT assay. This assay was repeated 3 times (*n* = 3).

The time required for the reaction to reach half of the maximum absorbance value, known as the halftime, was used to describe clotting over time for both RCT and FPA [70]. The clot density is presented as optical density (OD) at 30 min following the addition of CaCl_2_.

### 4.11. Statistical Analysis

The relationship between time (min) and the observed optical density (OD) values was estimated for the RCT and FPA assays using a nonlinear log-logistic regression model, parameterized according to Schabenberger et al. (1999) [71], to include a parameter estimating the clotting halftime with its 95% confidence interval in which the true population clotting halftime will lie with 95% confidence [72]. Hence different treatment conditions can be interpreted as having significantly different clotting halftimes when their confidence intervals do not overlap (α = 0.05). ANOVA models were fit to observed OD values, and mean comparisons were conducted using SAS PROC MIXED (2023, version 8) and the pdmix800 macro [73]. For the cathepsin assays using the fluorescence-labeled synthetic substrates, the observed fluorescence intensity values were first transformed by Log(OD + 1) to stabilize variability and mean comparison results were reported on the original data scale; statistics were obtained by back transforming the log(OD), mean, lower confidence limit, and upper confidence limit estimates [i.e., geometric mean(OD) = 10**(mean[log(OD)] [74].

## Figures and Tables

**Table 1:** Degradation of blood protein substrates by recombinant *O. ostertagi* cysteine proteases OoCBP1 and OoCBP2.

	OoCBP1							OoCBP2						
	bFg			bIgG		BSA	oHb	bFg			bIgG		BSA	oHb
	α	β	ɣ	H	L			α	β	ɣ	H	L		
pH 5.0	**++**	**++**	**++**	**-**	**-**	**+**	**+**	**++**	**++**	**++**	**+**	**-**	**++**	**++**
pH 5.0 + DTT	**++**	**++**	**++**	**+**	**-**	**++**	**++**	**++**	**++**	**++**	**++**	**+**	**++**	**++**
pH 7.4	**+**	**-**	**-**	**-**	**-**	**-**	**-**	**++**	**++**	**++**	**+**	**-**	**+**	**+**
pH 7.4 + DTT	**+**	**-**	**-**	**-**	**-**	**-**	**-**	**++**	**++**	**++**	**+**	**-**	**+**	**+**

The digestion was performed for 16 h at 37° C at pH 5.0 and 7.4 in the presence or absence of a reducing agent. bFg, bovine fibrinogen; bIgG, bovine immunoglobulin G; BSA, bovine serum albumin; oHb, ovine hemoglobin. α, β, and g represent 3 peptide chains of Fg; H, heavy chain of bIgG; L, light chain of bIgG. ++, complete degradation; +, partial degradation; -, no degradation.

## Data Availability

The original contributions presented in this study are included in the article/Supplementary Materials. Further inquiries can be directed to the corresponding author.

## Supplementary Materials

The following supporting information can be downloaded at: https://www.mdpi.com/article/10.3390/ijms27209219/s1.

## Abbreviations

OoCBPs	*Ostertagia ostertagi* cathepsin B-like proteases
BSA	Bovine serum albumin
bFg	Bovine fibrinogen
DTT	Dithiothreitol
E-64	l-trans-epoxysuccinyl-leucyl-amido (4-guanidino) butane
GI	Gastrointestinal
Hb	Hemoglobin
HEPES	4- (2-hydroxyethyl)-1-piperazineethanesulfonic acid
IgG	Immunoglobulin G
IACUC	Institutional Animal Care and Use Committee
L3, L4, L5	Third-, fourth-, and fifth-stage larvae
MES	2- (N-morpholino) ethanesulfonic acid
Ni-NTA	Nickel-Nitrilotriacetic Acid
OD	Optical density
PBS	Phosphate-buffered saline
RCT	Recalcification time
RCF	Relative centrifugal force
SDS-PAGE	Sodium Dodecyl Sulfate Polyacrylamide Gel Electrophoresis
Tris	Tris (hydroxymethyl) aminomethane
UCRP	Universal coagulation reference plasma
