# Supplementary material for: Host Protein-Degrading Cathepsin L-like Proteases of Ostertagia ostertagi
Source: Int J Mol Sci. 2026 Oct 17;27(20):9219. doi: 10.3390/ijms27209219 (PMC13705517; doi:10.3390/ijms27209219)
Supplement: Supplementary file 1 [file ijms-27-09219-s001.zip › ijms-4598497-supplementary.pdf]

A

```
CLUSTAL 2.1 multiple sequence alignment


OoCBP2          --MKYLVLALCVYLCRAASEAELLAALKAQQIPLEAQVLTGPPLVEYLRKNQKLFEVNSE
HcCBP2          MRYNVVALYICLSHVAFASQADVLAALKNQEIPLAAQVLSGEELVKYLQKNQNFFEADVT
OoCBP1          MKLLVLLFCIYLLQVINASEGDVLDALQAQRIPPEAQALSGEPLVEYLRKNQKLFEVEPN
HcCBP1          MNYFLTAFCIFLCRETATLNTEVLGDSKSDEIPLEAQMLTGEPLIRYLRENQNLFEVEP-
                       : : :     : : ::*   : :.**  ** *:*  *:.**::**::**.:

OoCBP2          PTPG-FKYKLMDLKFIDQNSNHVAEIVKDDDDVIPESFDARVQWANCSSVFHIRDQANCG
HcCBP2          PHSHNVQHKLMDLRFVNQNRKPVVENADDEDDDIPESFDARTHWANCTSLRHIRDQANCG
OoCBP1          PATHYYKKMLMDLEFIDPNHNTAPVDEADNGADIPESFDGRIQWAHCPSLTYIRDQSLCG
HcCBP1          -ARHDYKLKLMDIGLMDKNRKPVVENDGPDDD-IPESFDGRKVWSNCSSLSYIRDQSNCG
                      :  ***: ::: * : .      :.  ******.*  *::*.*: :****: **

OoCBP2          SCWAVSSASAMSDRVCIATQGAKQVLISDQDIVSCCTWCGYGCQGGWSIRAWYYFAEQGV
HcCBP2          SCWAVSTASALSDRICIASKGETQLHISSIDIVSCCKLCGYGCDGGWPIEAFDYFSRQGA
OoCBP1          SCWAISTAAAISDRLCIATNGSVKAEISANDILSCCTGCGWGCRGGWPVRAWRFFAKEGA
HcCBP1          SCWAVATASAISDRICILTKGAMQVTISATDILSCCEFCGFGCRGGSTIEAWNYFTEEGV
                ****:::*:*:***:** ::*  :  **  **:***  **:** ** .:.*: :*:.:*.

OoCBP2          VTGGNYNTKGSCRPYEIHPCGYHKDEPYYGECDDMAD-TPRCKRRCQLGYPKSYPSDKHY
HcCBP2          VTGGDYGSKDGCRPYPFHPCGHHGNDTYYGECPEDAS-TPKCRRRCQKGYKKSYKKDRTY
OoCBP1          VTGGNYGSKDCCRPYEIPPCGWHKGEPHYDCRALYKGGTPACEKKCQPGYNKNYTMDKFY
HcCBP1          VSGGNYGTKGCCQPYPLPPCGHHENETFYYECNKEAA-TPECQKRCHPGYRKLYRMDKFY
                *:**:*.:*. *:** : *** * .:..*         ** *.::*: ** * *  *: *

OoCBP2          GRTAYQLPMSVESIQREIMRNGPVVAGFTVYEDFAHYKGGIYKHTSGKKTGGHAVKVIGW
HcCBP2          GEDAYELPNSVKAIQKEIMENGPVVAVFTVYEDFSYYKKGIYVHIAGKARGAHAIKIIGW
OoCBP1          GKNGYFLPNSVIAIQREIMKNGPVVATFSVYEDFFHYRKGIYRRTAGKASGAHAAKIIGW
HcCBP1          GKGAYELPNSEKAIQREIMKHGPVVGMFRVFRDFYNYKSGVYKHTAGSQEGEHAVKIIGW
                *. .* ** *  :**:***.:****. * *:.**  *: *:* : :*.  * ** *:***

OoCBP2          GSEQKGSEKIPYWLVANSWHNDWGENGYFRIIRGINDCGFEERMVAGLVQG------
HcCBP2          GVENG----LPYWLIANSWHDDWGEQGLFRIVRGINECGIEGDVVAGHVQG------
OoCBP1          GKEGD----TPYWIIANSWHNDWGENGYFRMIRGINDCHIEMLVTAGLV--------
HcCBP1          GKEGE----TPYWLIANSWHHDWGENGFFKMLRGSNHCRIEELVVAGLVDNDVTRRL
                * *       ***::*****.****:* *:::** *.* :*  :.** *
```

B

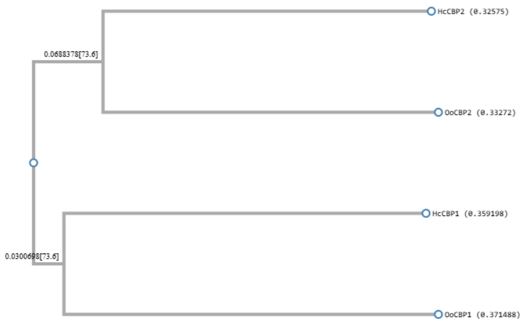

Figure S1. Multiple sequence alignment of OoCBP1 and OoCBP2 with *H. contortus* cathepsin-B-like proteins 1 and 2 (HcCBP1, HcCBP2). The conserved cysteine residues and signal peptide are highlighted; the 'haemoglobinase motif described for those cathepsins B, which are able to cleave hemoglobin, is indicated in bold face(A), and a phylogenetic tree (B) was generated from the resulting alignment.

A

```
CLUSTAL 2.1 multiple sequence alignment


OoCBP1          ----------------MKLLVLLFCIYLLQVINASEGDVLDALQAQRIPPEAQALSGEPL
TcCP            MELSEMSSFININHSLERLFAPLFCVYLLHVIYASEENVLDALRAQKILPEALALSGKSL
OoCBP2          ----------------MKYLVLALCVYLCR--AASEAELLAALKAQQIPLEAQVLTGPPL
HscatB          ----------------MWQLWASLCCLLVLANARSR-------------PSFHPLS-DEL
HscatL          -MNPTLILAAFCLGIASATLTFDHSLEAQWTKWKAMHNRLYG-MNEEGWRRAVWEKNMKM
HscatK          ----MWGLKVLLLPVVSFALYPEEILDTHWELWKKTHRKQYNNKVDEISRRLIWEKNLKY
                                   :                                   .

OoCBP1          VEYLRKNQKLFEVEPNPATHYYKKMLMDLEFIDPN--HNTAPVDEADNGADIPESFDGR-
TcCP            VEYLRKNQKLFEVELNPTKKFYKDMVMDPEFVSRDQYHNTAVLDESDNGDDIPESFDSR-
OoCBP2          VEYLRKNQKLFEVNSEPTPG-FKYKLMDLKFIDQNSNHVAEIVKDDD--DVIPESFDAR-
HscatB          VNYVNKRNTTWQAGHNFYNVDMSYLKRLCGTFLGGPKPPQRVMFTED--LKLPASFDAR-
HscatL          IELHNQEYREGKHSFTMAMNAFGDMTSEEFRQVMNGFQN---RKPRKGKVFQEPLFYEAP
HscatK          ISIHNLEASLGVHTYELAMNHLGDMTSEEVVQKMTGLKVPLSHSRSNDTLYIPEWEGRAP
                :.  . .                                       .

OoCBP1          --IQWAHCPSLTYIRDQSLCGSCWAISTAAAISDRLCIATNGSVKAEISANDILSCC-TG
TcCP            --IKWAHCPSLFYIRDQSLCGSCWAISTASAISDRLCIATNGTVKVHISGDDILSCC-RS
OoCBP2          --VQWANCSSVFHIRDQANCGSCWAVSSASAMSDRVCIATQGAKQVLISDQDIVSCC-TW
HscatB          --EQWPQCPTIKEIRDQGSCGSCWAFGAVEAISDRICIHTNAHVSVEVSAEDLLTCCGSM
HscatL          RSVDWREKGYVTPVKNQGQCGSCWAFSATGALEGQMFRKTG--RLISLSEQNLVDCSGPQ
HscatK          DSVDYRKKGYVTPVKNQGQCGSCWAFSSVGALEGQLKKKTG--KLLNLSPQNLVDCVSE-
                   .: .   :  :::*. ******..:. *:..::   *       :* :::: *

OoCBP1          CGWGCRGGWPVRAWRFFAKEGAVTGGNYGSKDCCRPYEIPPCGWHKGEPHYDCRALYKGG
TcCP            CGRGCNGGWPHTAWQFFAKEGAVTGGNYGSKDCCRPYEIPPCGWHKGEPHYDCRALYKGG
OoCBP2          CGYGCQGGWSIRAWYYFAEQGVVTGGNYNTKGSCRPYEIHPCGYHKDEPYYG-ECDDMAD
HscatB          CGDGCNGGYPAEAWNFWTRKGLVSGGLYESHVGCRPYSIPPCEHHVNGSRPP--CTGEGD
HscatL          GNEGCNGGLMDYAFQYVQDNGGLDS-------------------------------EES
HscatK          -NDGCGGGYMTNAFQYVQKNRGIDS-------------------------------EDA
                 . ** **    *: :   :  : .

OoCBP1          TPACEKKCQPGYNKNYTMDKFYGKNGYFLPNSVIAIQREIMKNGPVVATFSVYEDFFHYR
TcCP            TPACKRECQPGYSKNYTMDKYYGKNGYYLPN-VKTIQREIMRNGPVVAVYTVYQDFYHYT
OoCBP2          TPRCKRRCQLGYPKSYPSDKHYGRTAYQLPMSVESIQREIMRNGPVVAGFTVYEDFAHYK
HscatB          TPKCSKICEPGYSPTYKQDKHYGYNSYSVSNSEKDIMAEIYKNGPVEGAFSVYSDFLLYK
HscatL          YPYEATEESCKYNPKYSVANDTGFVDIPK-QEKALMKAVATVGPISVAIDAGHESFLFYK
HscatK          YPYVGQEESCMYNPTGKAAKCRGYREIPEGNEKALKRAVARVGPVSVAIDASLTSFQFYS
                 *      .  *  .    :  *                   .    .  :   .*  *

OoCBP1          KGIYRRTAG-KASGAHAAKIIGWGKE----GDTPYWIIANSWHNDWGENGYFRMIRG-IN
TcCP            KGIYKHTAG-QQTGGHAVKIIGWGEEKTGNKSIPYWIIANSWHNDWGENGYFRMIRG-IN
OoCBP2          GGIYKHTSG-KKTGGHAVKVIGWGSEQKGSEKIPYWLVANSWHNDWGENGYFRIIRG-IN
HscatB          SGVYQHVTG-EMMGGHAIRILGWGVEN----GTPYWLVANSWNTDWGDNGFFKILRG-QD
HscatL          EGIYFEPDCSSEDMDHGVLVVGYGFESTESDNNKYWLVKNSWGEEWGMGGYVKMAKDRRN
HscatK          KGVYYDESCNSDNLNHAVLAVGYGIQ----KGNKHWIIKNSWGENWGNKGYILMARNKNN
                 *:*      .    *.   :*:* :        :*:: ***  :**  *:. : :.  :

OoCBP1          DCHIEMLVTAGLV---------
TcCP            DCGLE-----------------
OoCBP2          DCGFEERMVAGLVQG-------
HscatB          HCGIESEVVAGIPRTDQYWEKI
HscatL          HCGIASAASYPTV---------
HscatK          ACGIANLASFPKM---------
                 * :
```

B

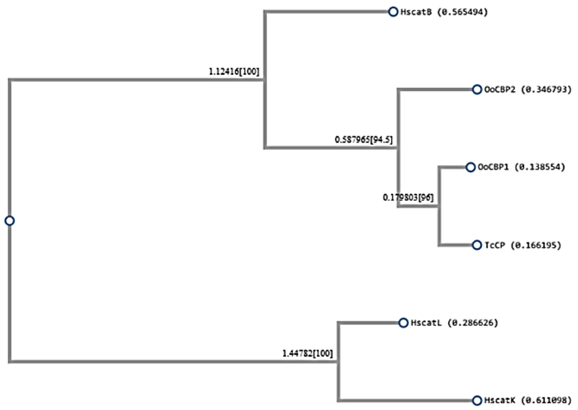

Figure S2. Protein sequences were aligned with Clustal W, and the conserved His, Cys, and Asn residues forming the catalytic active site are highlighted in yellow and conserved histidine residues within the potential occluding loop are highlighted in green (A). A phylogenetic tree (B) was generated from the resulting alignment. TcCP (PIO68319.1 papain family cysteine protease, partial [*Teladorsagia circumcincta*), HscatB (AAH95408.1 CatB [*Homo sapiens*]), HscatL(KAI4007581.1 cathepsin L [*Homo sapiens*]), HscatK(NP_000387.1 cathepsin K preproprotein [*Homo sapiens*]), OoCBP1 and OoCBP2 (*O. ostertagi* cathepsin-B-like proteins) (OoCBP1, accession Number PX866999; OoCBP2, accession Number PX867000).

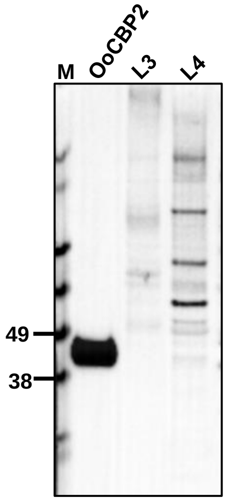

Figure S3. Western blot analyses of native OoCBP2 in Larval stages of *O. ostertagi.*
Lane M, protein molecular standards; L3 and L4, crude extract prepared from infective L3 and L4 worms, respectively.

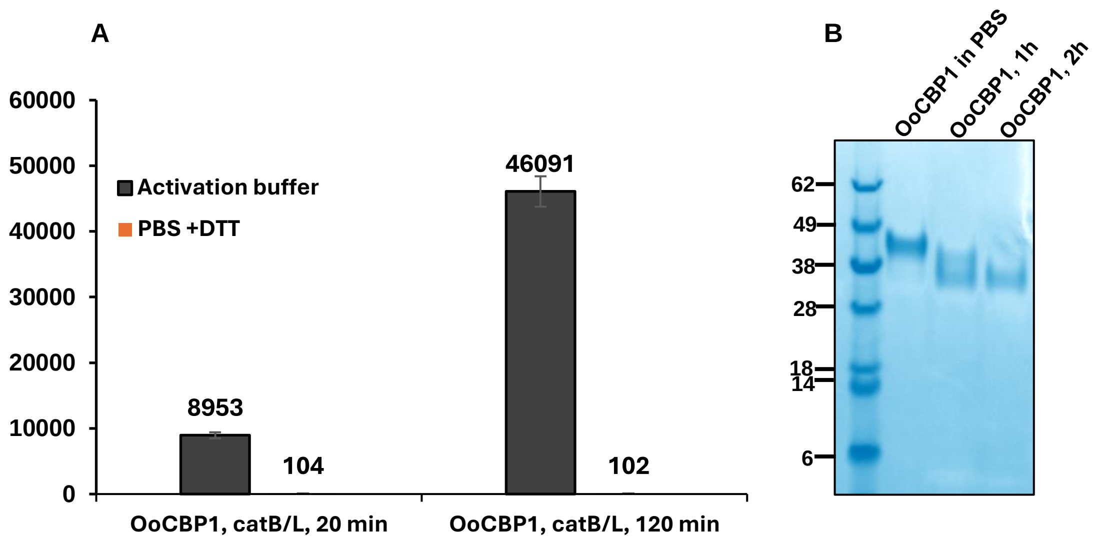

Figure S4. **Cathepsin-like activity of recombinant *O. ostertagi* cysteine protease (OoCBP1) was assayed using the synthetic substrate Z-Phe-Arg-AMC (catB/L-specific) at 37°C at pH 5.0 in 100 mM sodium acetate with 2 mM DTT and 2 mM EDTA following incubation for 20 min and 120 min (A), and SDS-PAGE analysis of OoCBP1 incubated in PBS for 2 h or in activation buffer for 1 h and 2 h (B). Lane M, protein molecular standards.**

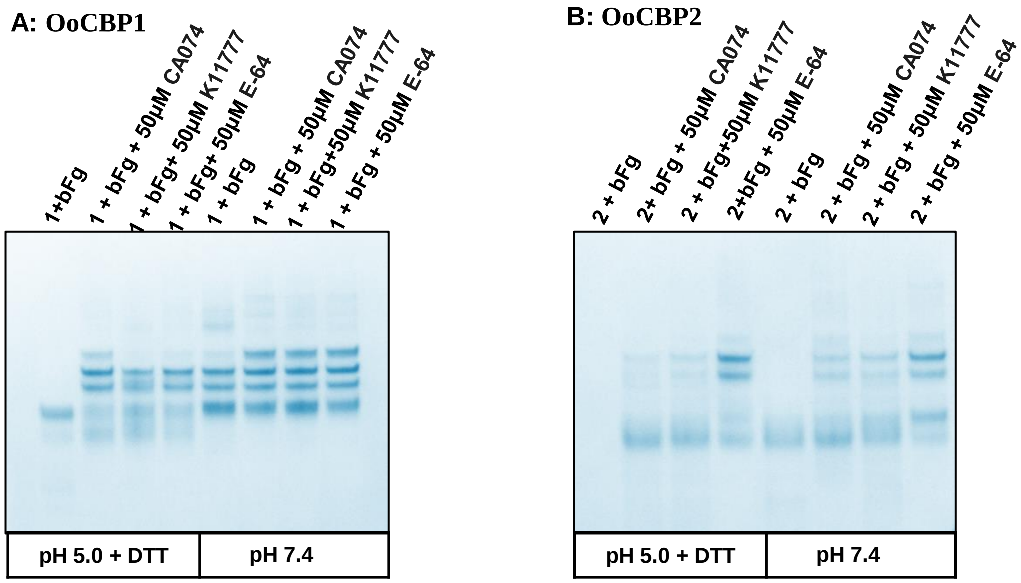

Figure S5. SDS-PAGE analysis of bFg degradation by OoCBP1(A) and OoCBP2(B) in the presence of protease inhibitors, including cysteine protease inhibitor (E-64), catB inhibitor(CA074), and catB/L inhibitor(K11777). OoCBPs were preincubated with 50 µM of each of the inhibitors for 5 min before adding bFg. The mixture was incubated for 16 h at 37 °C in PBS at pH 7.4 or in 20 mM MES buffer containing 5 mM DTT at pH 5.0. A and B. Lane M, protein molecular standards, OoCBP1 (1), OoCBP2 (2), bovine fibrinogen (bFg).

**Structure:**	**Chain:** A **Position**	**Distance (Å)**
Oocbp2_model	Between OD1 (A:ASN 313) and CB (A:CYS 119)	6.45
Oocbp2_model	Between CB (A:CYS 119) and ND1 (A:HIS 289)	3.13
Oocbp2_model	Between NE2 (A:HIS 289) and OD1 (A:ASN 313)	2.76
Oocbp1_model	Between CB (A:CYS 122) and OD1 (A:ASN 313)	6.38
Oocbp1_model	Between CB (A:CYS 122) and CE1 (A:HIS 293)	3.16
Oocbp1_model	Between NE2 (A:HIS 293) and OD1 (A:ASN 313)	2.73

Table S1. Distances between predicted active site residues measued using jFATCAT in Protean 3D, DNAstar version 18.1.1, DNASTAR, Inc. Madison, WI.

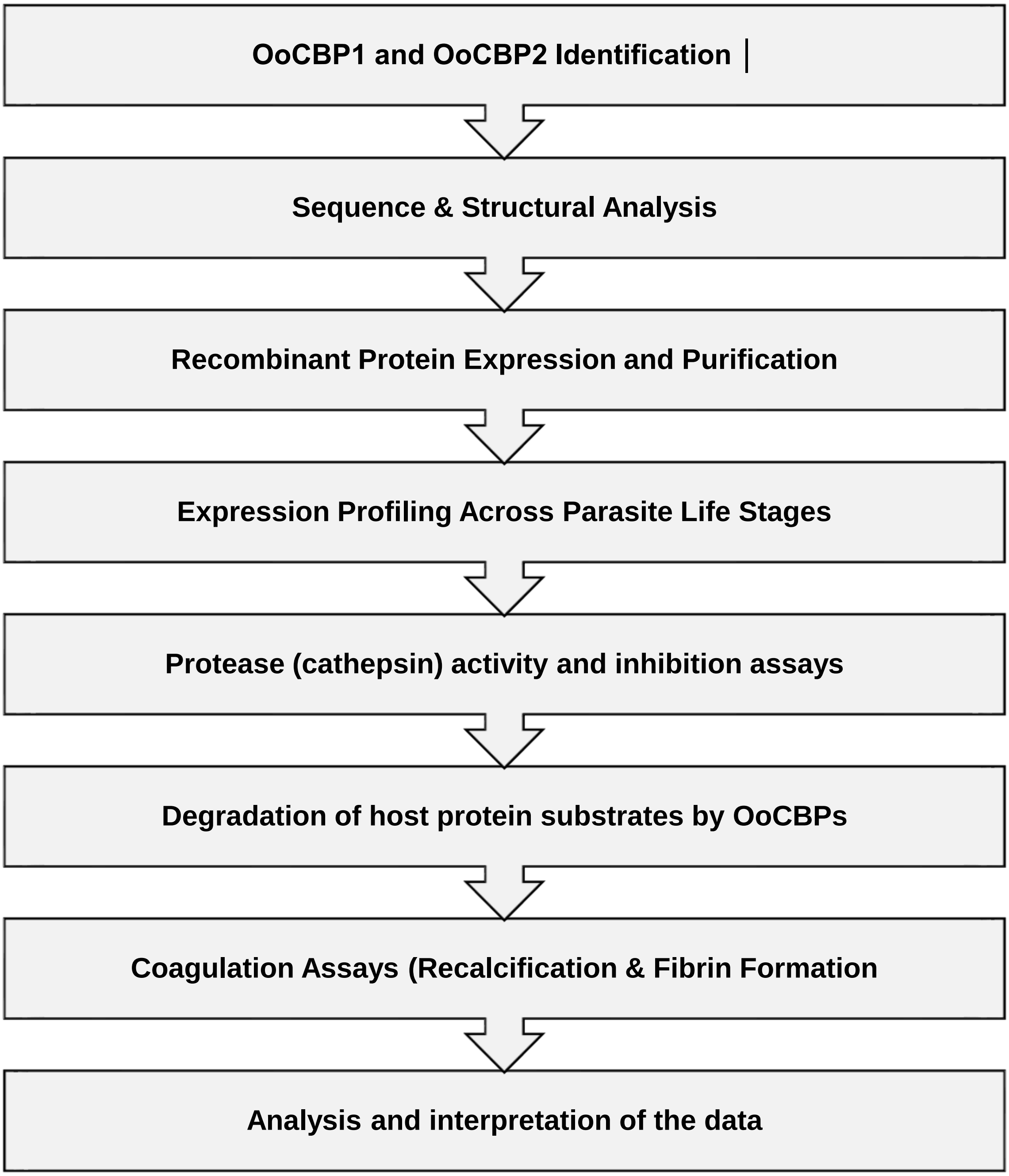

Figure S6. Experimental workflow for the characterization of the cathepsin B-like proteases OoCBP1 and OoCBP2 from *Ostertagia ostertagi*. The study included protein identification, sequence and structural analyses, recombinant protein production, expression profiling across parasite life stages, enzymatic characterization, host protein degradation assays, coagulation assays, and Analysis and interpretation of the data.
